# The exonuclease Xrn1 activates transcription and translation of mRNAs encoding membrane proteins

**DOI:** 10.1038/s41467-019-09199-6

**Published:** 2019-03-21

**Authors:** Bernat Blasco-Moreno, Leire de Campos-Mata, René Böttcher, José García-Martínez, Jennifer Jungfleisch, Danny D. Nedialkova, Shiladitya Chattopadhyay, María-Eugenia Gas, Baldomero Oliva, José E. Pérez-Ortín, Sebastian A. Leidel, Mordechai Choder, Juana Díez

**Affiliations:** 1 Virology Unit, Department of Experimental and Health Sciences, Universitat Pompeu Fabra, Barcelona, 08003 Spain; 2 Departments of Genetics, Biochemistry and Molecular Biology, and ERI Biotecmed, Facultad de Biología, Universitat de València, Burjassot, E-46100 Spain; 3 Max Planck Research Group for RNA Biology, Max Planck Institute for Molecular Biomedicine, 48149 Muenster, Germany; 4 Department of Molecular Microbiology, Rappaport Faculty of Medicine, Technion-Israel Institute of Technology, Haifa, 31096 Israel; 5 Laboratory of Structural Bioinformatics (GRIB), Department of Experimental and Health Sciences, Universitat Pompeu Fabra, Barcelona, 08003 Spain; 6 Department of Chemistry and Biochemistry, University of Bern, Bern, 3012 Switzerland; 7 Present Address: Mechanisms of Protein Biogenesis Laboratory, Max Planck Institute of Biochemistry, 82152 Martinsried, Germany

## Abstract

The highly conserved 5’–3’ exonuclease Xrn1 regulates gene expression in eukaryotes by coupling nuclear DNA transcription to cytosolic mRNA decay. By integrating transcriptome-wide analyses of translation with biochemical and functional studies, we demonstrate an unanticipated regulatory role of Xrn1 in protein synthesis. Xrn1 promotes translation of a specific group of transcripts encoding membrane proteins. Xrn1-dependence for translation is linked to poor structural RNA contexts for translation initiation, is mediated by interactions with components of the translation initiation machinery and correlates with an Xrn1-dependence for mRNA localization at the endoplasmic reticulum, the translation compartment of membrane proteins. Importantly, for this group of mRNAs, Xrn1 stimulates transcription, mRNA translation and decay. Our results uncover a crosstalk between the three major stages of gene expression coordinated by Xrn1 to maintain appropriate levels of membrane proteins.

## Abstract (web summary)

The exonuclease Xrn1 mediates crosstalk between transcription and mRNA decay in yeast. Here the authors demonstrate that Xrn1 promotes translation of mRNAs encoding membrane proteins, coupling transcription, translation, and mRNA decay.

## Introduction

Proper tuning of protein levels under normal and perturbed conditions requires precise regulations at different stages of gene expression. These stages, classically considered isolated because of their different spatial and temporal incidence, are indeed interconnected. A major crosstalk between transcription and decay is mediated by Xrn1^1–3^, a highly conserved exoribonuclease, which is the 5ʹ–3ʹ messenger RNA (mRNA) degradation enzyme in the cytoplasm^4,5^. Within the deadenylation-dependent decay pathway, Xrn1 forms a complex interaction network with the Dcp1/Dcp2 decapping enzyme and decapping activators, such as Lsm1–7, Pat1, and Dhh1/DDX6^6,7^. At least some of the degradation activity of Xrn1 occurs co-translationally^8^. Xrn1 further participates in the decay of mRNAs after internal cleavage and in the cytoplasmic mRNA surveillance system that degrades aberrant mRNAs^7^. Moreover, Xrn1 directs degradation of long non-coding RNAs and hypomodified transfer RNA (tRNA), as well as maturation of ribosomal RNAs (rRNAs)^7,9–12^.

Remarkably, besides these exonucleolytic functions, Xrn1 acts as a transcriptional activator. Xrn1, together with other components of the deadenylation-dependent mRNA decay pathway, shuttles between the cytoplasm and the nucleus, where they bind to transcription start sites and directly stimulate transcription initiation and elongation of many yeast genes^1^. The functions of Xrn1 in both cellular compartments are linked. Shuttling of Xrn1 and other decay factors to the nucleus depends on the proper exoribonucleolytic activity of Xrn1. By connecting mRNA synthesis to decay, Xrn1 maintains mRNA homeostasis, as defects in 5ʹ–3ʹ mRNA decay are buffered by reductions in mRNA synthesis. Whether Xrn1 functions in other stages of gene expression has not been considered.

Here, we show that Xrn1 acts as a translational modulator. This unanticipated function is restricted to specific groups of genes enriched in distinct GO terms. Interestingly, Xrn1 activates both translation of mRNAs encoding membrane proteins and their localization at the endoplasmic reticulum, the translation compartment of membrane proteins. These mRNAs contain long and highly structured 5ʹUTRs. A physical and functional interaction of Xrn1 with the translation initiation factor eIF4G is required for translational activation, likely to overcome these unfavorable contexts for translation initiation. Remarkably, the group of mRNAs that depend on Xrn1 for translation highly depend on Xrn1 for transcription and decay. Moreover, these three functions of Xrn1 are linked. Our results show a coordinated control of the three main stages of gene expression by Xrn1 to maintain proper homeostasis of membrane proteins. This coordination may be important to prevent their toxic aggregation.

## Results

### Xrn1 drives translation of Brome mosaic virus RNA2

**Fig. 1:**
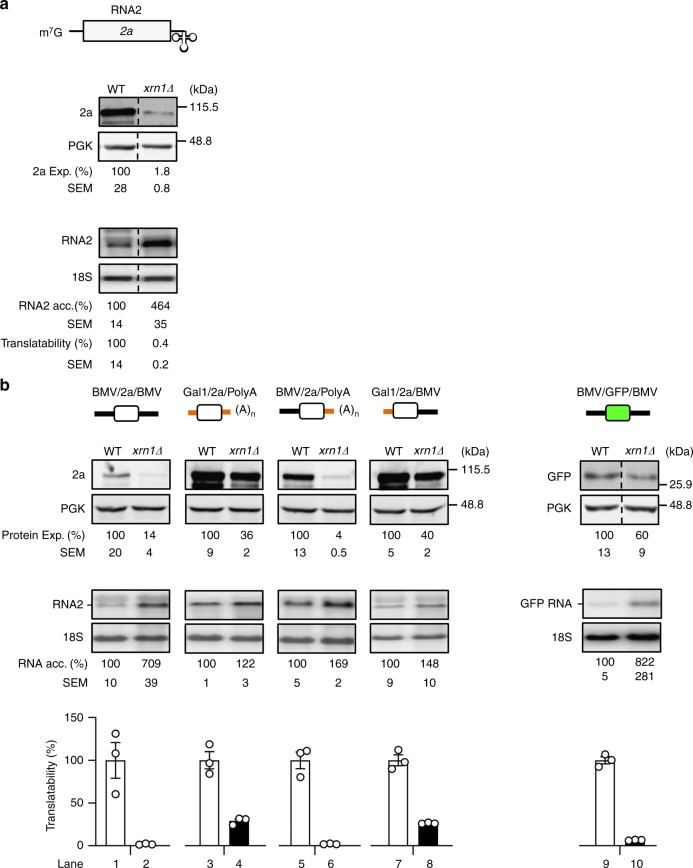
Xrn1 drives translation of BMV RNA. **a** Xrn1 depletion inhibits BMV RNA2 translation. Simultaneously exposed western blot and northern blot panels showing steady-state levels of viral protein 2a and RNA2. **b** The BMV RNA2 5ʹUTR and CDS confer dependence on Xrn1 for translation. Black solid lines represent viral UTRs and orange lines *GAL1* mRNA 5ʹ and *ADH1* 3ʹUTRs. The white and green boxes represent 2a and GFP CDSs, respectively. Throughout this study, BMV RNA2 was expressed from a plasmid by the *GAL1* promoter, PGK protein, and 18 S RNA were used as loading controls for western and northern blots, respectively. Values denote expression relative to WT, taken arbitrarily as 100% and are calculated from *n* = 3 independent colonies and expressed as mean ± SEM. Dotted lines represent a separation of the shown samples in the same membrane. Open circles indicate the individual data points. Source data are provided as a Source Data file

Given the multifunctional nature of Xrn1 and its association with translating mRNAs during co-translational decay, we examined whether Xrn1 in addition regulates translation. As a first approach, we used the Brome mosaic virus (BMV)/yeast system. The ability of the plant BMV RNA2 genome to translate in yeast is a useful tool to identify and characterize specialized translational control mechanisms of host mRNAs^13,14^. The 5ʹcapped BMV RNA2 contains a tRNA-like structure instead of a poly(A) tail at the 3´end. Its long and structured 5ʹUTR and coding sequence (CDS) contain *cis*-sequences involved in translational control^13,15^. When expressed in yeast, RNA2 is recognized by the ribosomes to translate the viral 2a polymerase. The complete BMV lifecycle occurs within the cytoplasm. However, as we were interested in identifying translational control mechanisms affecting cellular mRNAs, we expressed the RNA2 from a cellular promoter. We transformed wild-type (WT) yeast and an isogenic *XRN1* deletion strain (*xrn1∆*) with a plasmid expressing BMV RNA2 by the *GAL1* promoter, whose transcription is activated by Xrn1. Interestingly, whereas the steady-state level of the viral RNA2 was increased in *xrn1∆* cells, expression of the 2a protein was substantially decreased (Fig. 1a). Translatability of RNA2 (change in 2a protein level divided by change in RNA2 level) in *xrn1∆* was only 0.4% of that in the WT. In *xrn1∆* cells the majority of mRNA molecules are capped^16^ and a major fraction of uncapped mRNAs is associated to polyribosomes. Similarly, we found that most of RNA2 molecules (77%) are capped (Supplementary Fig. 1). In our calculations, we used total RNA2 instead of capped RNA2 as the difference in the obtained values is minimal. Translatability of RNA2 in *xrn1∆* cells when considering only capped RNA2 is 0.5% instead of 0.4%. To determine quantitatively whether the Xrn1 effect on 2a protein levels involves protein degradation, we fused 2a to Renilla luciferase (2a-Rluc). Turnover of 2a-Rluc was determined by blocking translation with cycloheximide and measuring luciferase activity thereafter. Whereas translatability of the chimeric mRNA was affected by deleting *XRN1* (Supplementary Fig. 2a), the 2a-Rluc protein turnover was not (Supplementary Fig. 2b). We conclude that Xrn1 promotes translation of RNA2.

To assess which regions of BMV RNA2 confer Xrn1-dependence for translation, we replaced different RNA2 sequences and quantified 2a and RNA2 levels in the presence or absence of Xrn1. The 5ʹUTR was replaced by the *GAL1* 5ʹUTR and the non-polyadenylated 3ʹUTR by the polyadenylated 3ʹUTR of the *ADH1* transcript^14^ (Fig. 1b). These changes affected both protein and RNA levels. Replacement of RNA2 3ʹUTR with *ADH1* 3ʹUTR had no effect on translatability (Fig. 1b, lane 6). However, the 5ʹUTR played an important role on the capacity of Xrn1 to affect translatability (Fig. 1b, lane 8). Replacement of RNA2 CDS with GFP CDS had a modest effect on translatability (Fig. 1b, lane 10). Taken together, results in Fig. 1b indicate that the 5ʹ UTR is the most Xrn1-responsive region in RNA2.

**Fig. 2:**
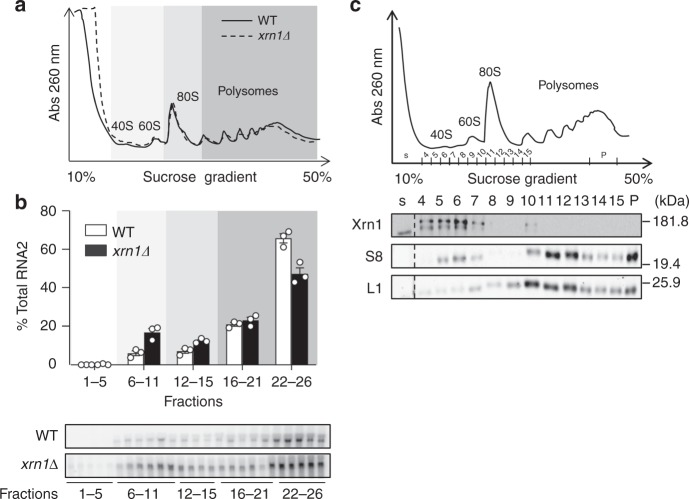
Xrn1 depletion shifts RNA2 toward single ribosomal subunits fractions. **a** ultraviolet (UV) absorbance rRNA profile at 260 nm of an extract from WT and *xrn1∆* cells expressing RNA2 after sedimentation on a 10 to 50% sucrose gradient. **b** Depletion of Xrn1 shifts BMV RNA2 toward monosomal fractions. Upper panel: distribution of normalized BMV RNA2 accumulation across the profiles. Fractions were grouped into free (1–5), single ribosome subunits (6–11), monosomes (12–15), light polysomes (16–21), and heavy polysomes (22–26). RNA was quantified by northern blot. Results represent averages of *n* = 3 biological replicates. Error bars represent SEM. Open circles indicate the individual data points. Lower panel: representative northern blots. **c** Xrn1 cofractionates with free 40s subunits in polysome-profiling analysis. Top panel: UV absorbance rRNA profile at 260 nm of an extract from WT cells. Lower panel: Fractions were TCA-precipitated and analyzed by western blot. Specific antibodies detecting Xrn1p, S8 protein (small ribosomal subunit) and L1 protein (large ribosomal subunit) were used. S lane corresponds to soluble proteins not associated to ribosome subunits and P lane to a pool of three fractions corresponding to polysomes. Source data are provided as a Source Data file

To investigate which step of BMV RNA2 translation is stimulated by Xrn1, we performed polysome-profiling analyses in WT and *xrn1∆* cells expressing RNA2. Consistently with previous studies^17^, the global rRNA profile, indicative of the global translation, was only mildly affected in *xrn1∆* (Fig. 2a). Northern blot analyses along the polysome profile showed that deletion of *XRN1* shifted RNA2 toward monosomal, 60s and 40s fractions (Fig. 2b), suggesting a role of Xrn1 in translation initiation. In agreement with the known dependence of polysomes on the presence of Mg^2+^, EDTA treatment shifted BMV RNA2 from heavy polysomes to lighter fractions (Supplementary Fig. 3). To further strengthen the link of Xrn1 to translational control, we examined whether Xrn1 co-sediments with ribosomes in polysome profiling. We observed an enrichment of Xrn1 in fractions corresponding to 40s subunits (Fig. 2c). Collectively, these results indicate that Xrn1 is required for efficient translation of RNA2, likely at early events of the translation initiation step.

### The effect of Xrn1 on BMV RNA2 translation is specific

**Fig. 3:**
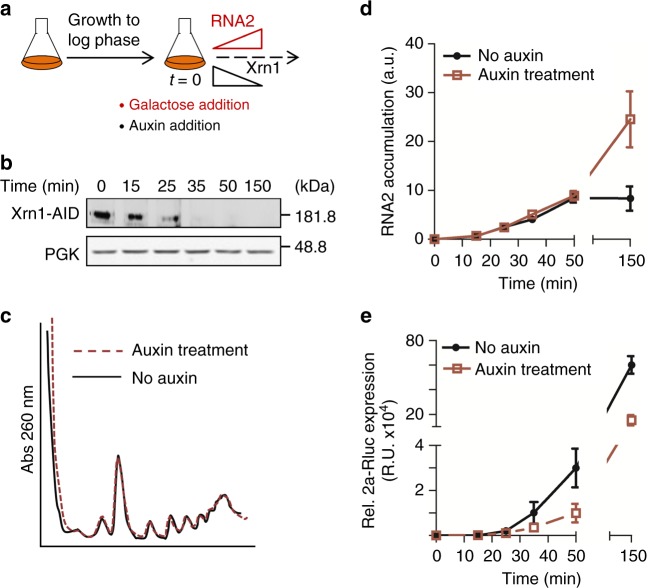
Xrn1 depletion leads to immediate defects on viral RNA2 translation. **a** Scheme of the experimental set-up to simultaneously deplete Xrn1-AID and express viral 2a-Rluc. **b** Expression of Xrn1-AID upon addition of auxin to the media. **c** UV absorbance profile at 260 nm of an extract from the Xrn1-AID strain before and after 35 min of auxin addition. **d** Relative RNA2-Rluc RNA accumulation, obtained by quantifying RNA level by RT-qPCR (arbitrary units (a.u)) and **e** Relative 2a-Rluc protein expression (R.U.) before and after galactose and auxin addition. Results represent averages obtained from *n* = 3 biological replicates. Error bars represent SEM. Source data are provided as a Source Data file

Stably deleting *XRN1* can lead to selection of adaptive secondary mutations that might cause indirect effects. To overcome this potential limitation, we fused Xrn1 to an auxin-inducible degron (AID) that induces rapid degradation of Xrn1 and measured the immediate effects^18^. AID-tagging of Xrn1 did not significantly affect its function in BMV RNA2 translation (Supplementary Fig. 4). WT cells carrying AID-tagged *XRN1* in its natural genomic locus and a plasmid expressing RNA2-Rluc from a *GAL1* promoter were grown in raffinose to logarithmic phase. Addition of galactose and auxin resulted in simultaneous induction of BMV RNA2-Rluc transcription and Xrn1-AID depletion (Fig. 3a). Xrn1-AID protein levels decreased upon addition of auxin and were no longer detected after 35 min (Fig. 3b) while global translation was not affected at this time-point (Fig. 3c). Induction kinetics of RNA2-Rluc was comparable in WT and *xrn1*∆ (Fig. 3d), probably due to the compensatory effect of transcription and decay of this transcript. In contrast, the level of 2a-Rluc protein, which reflects mainly translation at these early time-points after galactose induction, was reduced in auxin-treated cells (Fig. 3e). The effect of Xrn1-AID depletion was observed already at early time-points after auxin addition, indicating that depletion of Xrn1 inhibits RNA2 translation immediately and that an indirect effect is unlikely.

**Fig. 4:**
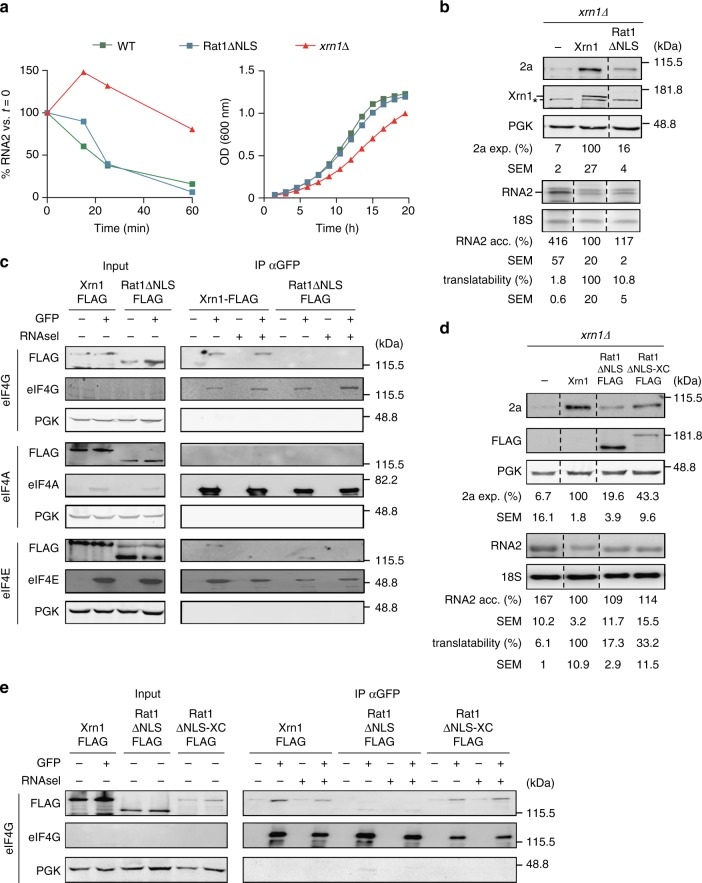
Xrn1-dependence for translation is specific and mediated by the interaction with eIF4G. **a** Rat1∆NLS fully replaces Xrn1 in mRNA decay and cell proliferation. *xrn1∆* cells expressing BMV RNA2 under *GAL1* promoter and either WT Xrn1, Rat1∆NLS or an empty plasmid were grown in galactose. Left, transcription of RNA2 was shut-off upon glucose addition and BMV RNA2 stability was determined by monitoring RNA2 levels by northern blot analysis at various time-points post-glucose addition. A representative example out of three replicates is shown. Right, growth curves in galactose media. Results represent averages obtained from three replicates. **b** Rat1∆NLS does not replace Xrn1 in RNA2 translation. Western blot (upper panel) and northern blot (lower panel) analysis showing steady-state levels of viral protein 2a and viral RNA2 in *xrn1∆* cells expressing Xrn1 or Rat1∆NLS. Asterisk points at a non-specific band. Quantifications are relative to *xrn1∆* transformed with WT Xrn1 plasmid. Results represent averages of *n* = 3 biological replicates. **c** Xrn1 interacts with eIF4G. Western blot analysis of immunoprecipitation assays. Xrn1-FLAG and Rat1∆NLS-FLAG proteins were expressed in yeast strains expressing either eIF4G-GFP, eIF4A-GFP, or eIF4E-GFP fusion proteins. As a control, the functionality of GFP-fused strains was assessed (Supplementary Fig. 5). Immunoprecipitations were carried out with GFP-trap beads with extracts treated ( + ) or not treated (−) with RNase A. Expression levels of eIF4G, eIF4A, and eIF4A were detected by anti-GFP antibody. **d** Expression of Rat1∆NLS-XC rescues BMV RNA2 translation. Western blot (upper panel) and northern blot (lower panel) analysis. Results represent averages of three replicates. Expression levels of flag-tagged Xrn1, Rat1∆NLS, and Rat1∆NLS-XC were analyzed by western blot (Supplementary Fig. 6). **e** Interaction with eIF4G studied by co-immunoprecipitation analyses. Source data are provided as a Source Data file

Next, by replacing Xrn1 with its nuclear paralog Rat1 we explored whether the positive role of Xrn1 in translation is specific. When forced to localize in the cytoplasm by deleting its nuclear localization signal (NLS), Rat1∆NLS functionally replaces Xrn1^19^. Accordingly, expression of Rat1∆NLS in *xrn1∆* cells fully rescued viral RNA2 degradation and cellular growth (Fig. 4a). In contrast, Rat1∆NLS did not efficiently rescue translation of BMV RNA2, since, upon addition of Rat1∆NLS to *xrn1∆* cells, the expression of protein 2a was only marginally recovered (Fig. 4b). Hence, viral RNA2 translation requires an Xrn1-specific function, not simply its 5ʹ to 3ʹ exonuclease activity per se.

As our polysome-profiling results suggested a role of Xrn1 in translation initiation (Fig. 2b), we examined whether Xrn1 interacts with the eIF4F complex, a key component of the translation initiation machinery that binds to capped mRNA and mediates its interaction with the 43S pre-initiation complex^20^. This complex consists of the RNA helicase eIF4A, the cap-binding proteins eIF4E and the large eIF4G scaffold protein. We carried out immunoprecipitation assays using yeast strains carrying functionally validated genomic GFP-tag fusions of eIF4G, eIF4A, or eIF4E transformed with plasmids expressing FLAG-tag fusions of Xrn1 or Rat1∆NLS (Fig. 4c and Supplementary Figs. 5 and 6). Remarkably, Xrn1, but not Rat1∆NLS, co-immunoprecipitated with eIF4G in an RNase-resistant manner. Neither Xrn1 nor Rat1∆NLS interacted with eIF4A while RNase-sensitive interactions with eIF4E were detected for both Xrn1 and Rat1∆NLS. Thus, Xrn1, but not Rat1∆NLS, interacts with eIF4G. To test whether the ability of Xrn1 to interact with eIF4G is functionally linked to its role in translation we designed a gain-of-function experiment. The sequence and folding of the N-terminal exonuclease domains of Rat1 and Xrn1 are very similar. However, Rat1 lacks an unstructured C-terminal domain present in Xrn1 that serves as an interaction platform in higher eukaryotes^21^. We used structural modeling to generate a chimera between the Rat1∆NLS N-terminal domain and the Xrn1 C-terminal tail (Rat1∆NLS-XC, Supplementary Fig. 7a, b) and tested its expression (Supplementary Fig. 6) and functionality (Supplementary Fig. 7c). As found for Rat1∆NLS, the chimeric protein rescued cellular growth in *xrn1∆* cells indicating that the fusion of the Xrn1 C-terminal tail does not compromise the global function of Rat1∆NLS. Expression of Rat1∆NLS-XC resulted in a twofold increase of viral 2a expression when compared to Rat1∆NLS (Fig. 4d) while the steady-state levels of RNA2 were similar. Notably, the Rat1∆NLS-XC chimera concomitantly gained interaction with the translation initiation factor eIF4G (Fig. 4e). Consistently, in comparison to wild-type Xrn1, expression of Rat1ΔNLS shifted RNA2 from light polysomes to 40s and 60s fractions in polysome-profiling experiments. This shift was partially abrogated when Rat1ΔNLS-XC was expressed (Supplementary Fig. 8a). Overall our data suggest a specific function of Xrn1 in translation mechanistically linked to its C-terminal domain and the ability to interact with eIF4G.

### Xrn1 drives translation and localization of secretome mRNAs

**Fig. 5:**
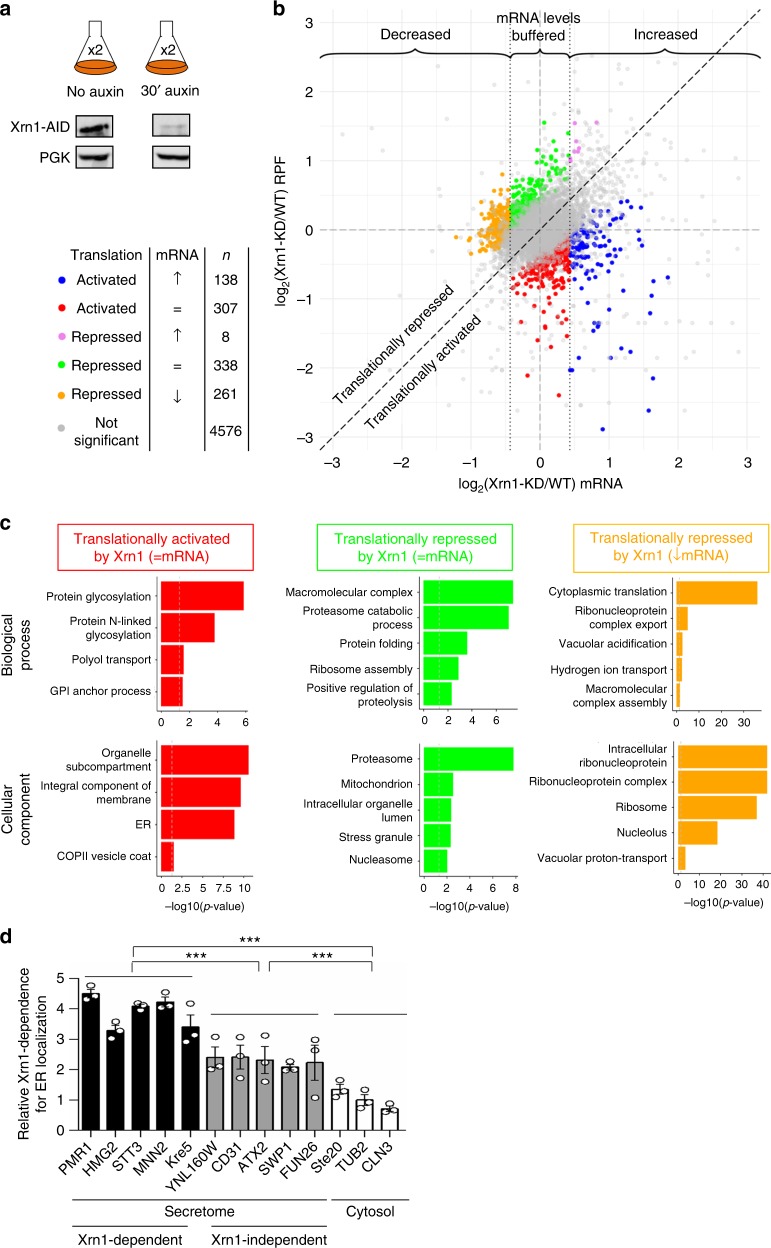
Xrn1 acts as a translational regulator of cellular mRNAs. **a** Experimental set-up used for ribosome-profiling analysis. Two duplicates for each condition (no auxin and 30 min with auxin) were included (*n *= 2). Western blot analysis corroborated Xrn1 depletion upon auxin addition. **b** Analysis of the results from the RNAseq and RPF libraries, comparing untreated cells (WT) to auxin-treated cells (Xrn1-KD). Log_2_ fold changes in mRNA abundance and ribosome occupancy (RPF) are represented. Vertical dashed lines in black depict the thresholds chosen to consider mRNA levels as stable/buffered ((log_2_ fold change) < 0.433); see ref. ^13^). A perfect correlation between changes in mRNA and ribosome occupancy levels is indicated by a dashed diagonal line. Gray dots correspond to genes with no significant change in translational control (Riborex FDR ≥ 0.05). Colored dots correspond to genes that show significant changes in translational regulation upon Xrn1 depletion (Riborex FDR < 0.05). These genes are classified into five different subgroups according to their behavior in terms of mRNA and RPF changes. **c** Gene ontology enrichment analysis (GSEA) of transcripts translationally regulated by Xrn1, focusing on Biological Process and Cellular Component. Colors correspond to the same groups of genes as described in Fig. 5b. At most five significant terms after Revigo redundancy removal are depicted (ordered by *p*-value). **d** Xrn1-dependence for translation correlates with Xrn1-dependence for ER localization. The *y*-axis indicates the ER localization:cytosol-localization in WT (no auxin) related to Xrn1-depleted cells (30 min of auxin treatment). The ratio of specific mRNAs between supernatant and membrane fractions was calculated in WT and *xrn1∆* conditions. Xrn1-dependence for ER localization was calculated by dividing [(cc/cm) *xrn1∆*]/[(cc/cm) WT] (cc = conc. in the cytosol fraction; cm = concentration in the membrane fraction). The values were represented relative to *TUB2*, which was set to 1. Open circles indicate the individual data points. Values are the mean of *n* = 3 assays ± SEM. Statistical significance was calculated using a Student’s *t*-test (***p-value < 0.001). Source data are provided as a Source Data file

Our results using the BMV RNA2 model in yeast prompted us to investigate whether Xrn1 also regulates translation of cellular mRNAs. We used the Xrn1-AID degron system to avoid adaptive effects and studied genome-wide translational changes using ribosome-profiling. This method is based on the isolation and deep-sequencing of ribosome-protected fragments (RPFs) and parallel transcriptome analysis^22^. Ribosome-profiling was performed on samples before and after 30 min of auxin treatment (Fig. 5a). Replicates of the RPF and RNAseq libraries clustered in principal-component analyses (Supplementary Fig. 9). To identify genes showing changes of translational efficiency upon degron-mediated Xrn1 knock-down (Xrn1-KD), we used the Riborex R-package^23^, which assesses whether changes of ribosome occupancy could be explained by changes of the corresponding mRNA. Genes were plotted according to their log_2_-fold changes in mRNA abundance and ribosome occupancy (RPF) (Fig. 5b). A majority of genes showed no significant changes in translational efficiency. Strikingly, we identified a specific set of genes translationally activated (445) or repressed (597) by Xrn1. Genes showing significant alterations of translational efficiency (FDR < 0.05) were grouped according to their relative mRNA and RPF log_2_ fold changes (log_2_FC, obtained via DESeq2). If mRNA levels changed within a range of approximately ± 35%, they were considered as stable/buffered (similar to ref. ^13^), (Fig. 5b and Supplementary Data 1). We classified genes with reduced translatability upon Xrn1 depletion (Xrn1-KD) as translationally activated by Xrn1 whereas translationally repressed genes correspond to those with increased translatability. Using these criteria, we obtained five different classes of genes that were subsequently tested for functional enrichment via gene ontology (GO) terms. Importantly, three of these groups showed distinct and highly significant functional enrichments. (I) translationally activated genes whose mRNA levels are buffered (red) were enriched for genes related to glycosylation, membranes and ER, (II) translationally repressed genes with buffered mRNA levels (green) were related to proteasome function and protein folding, and (III) translationally repressed genes with decreased mRNA levels (orange) showed enrichment for cytoplasmic translation and ribosomes (Fig. 5c and Supplementary Data 2). These results revealed that Xrn1-dependent regulation characterizes at least three different groups of genes, which are defined by different behaviors in RNA steady-state levels and translation, and are enriched for different cellular functions.

The absence of Xrn1 can lead to accumulation of uncapped mRNAs that are normally degraded by Xrn1 (see Supplementary Fig. 1), which might affect our results. However, as we observe for RNA2, most of the mRNAs are capped in *xrn1Δ* cells^16^. The minor fraction of deadenylated and uncapped mRNAs is mainly associated to polyribosomes^16^ and thus are captured by the ribosome-profiling libraries but are depleted from the oligo(dT)-selected RNAseq libraries. Thus, the activating effects of Xrn1 on the translationally activated mRNAs would be slightly higher than our estimates, while the repressing effects on the translationally repressed mRNAs would be slightly lower.

The cytosol and the ER represent distinct biological environments for translation with different regulatory factors. As membrane proteins are translated and glycosylated at the ER, we questioned whether Xrn1-dependence for translation is linked to a possible role of Xrn1 in localizing the affected mRNAs to the ER. Based on our Ribosome-profiling data, we selected three groups of transcripts: (i) transcripts encoding membrane proteins that depend on Xrn1 for translation and, as controls, (ii) transcripts encoding membrane proteins that do not depend on Xrn1 for translation, and (iii) transcripts encoding cytosolic proteins. Next, we isolated ER membranes from the cytosol and quantified the relative amounts of the selected transcripts associated to ER and cytosol in WT as compared with that in Xrn1-KD cells (Fig. 5d). Transcripts that depend on Xrn1 for translational activation exhibited a fourfold increase in ER localization when Xrn1 was present while transcripts that do not depend on Xrn1 for translation exhibited a twofold increase. No such effect was observed for cytosolic transcripts. Membrane proteins (as defined in ref. ^24^) were enriched among the Xrn1-dependent transcripts of our ribosome-profiling data as Xrn1-activated transcripts were 2.09 times more likely to be part of the membrane compared to transcripts that do not depend on Xrn1 for translation. In contrast, transcripts that were translationally repressed upon Xrn1-KD were devoid of membrane genes (Supplementary Data 3). Considering that our current subset of Xrn1-activated genes represents a stringent selection of Xrn1-dependent events, our analysis might miss transcripts affected by Xrn1 depletion (false negatives). Based on the observed behavior of membrane genes among transcripts that are translationally controlled by Xrn1, many of these false negatives can be anticipated to be membrane genes as well. This might explain the twofold increase in ER localization observed in mRNAs encoding membrane proteins classified as not dependent on Xrn1.

We conclude that Xrn1-KD cells are defective in recruiting mRNAs to the ER while the extent of this defect correlates with the capacity of Xrn1 to activate translation. As this defect was observed shortly after Xrn1 depletion, it is unlikely an indirect effect of the absence of Xrn1. The described routes of targeting mRNAs to the ER for translation include signal recognition particle (SRP)-dependent and -independent pathways. Based on previous studies^24,25^ we found that both SRP-dependent and -independent transcripts are similarly represented in the transcripts encoding membrane proteins whose translation is activated by Xrn1. This argues in favor of Xrn1 functioning along both routes.

### Xrn1-activated mRNAs have long and structured 5ʹUTRs

**Fig. 6:**
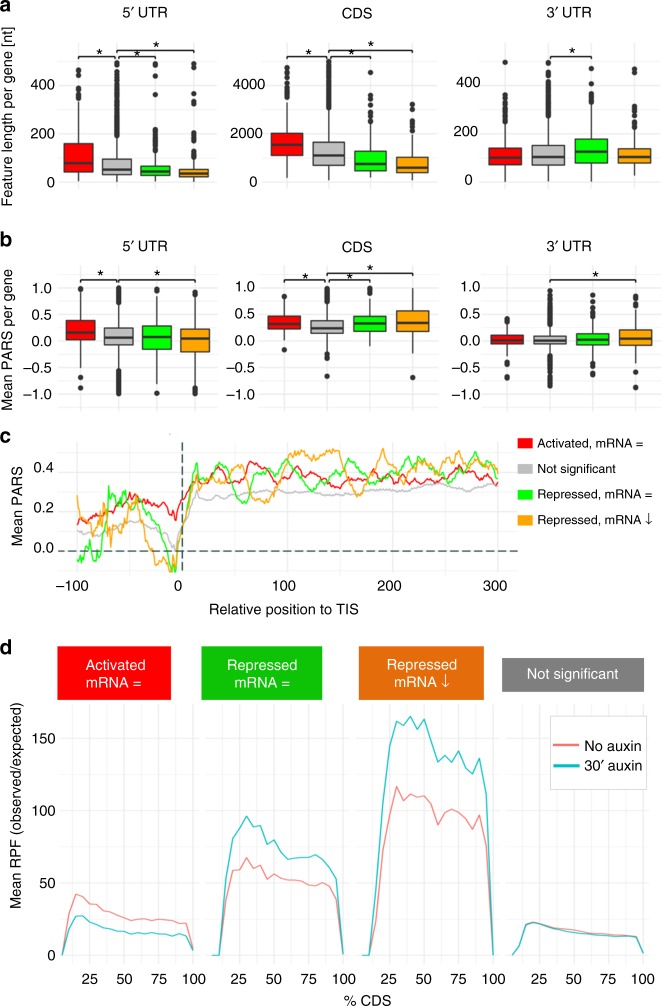
Features of mRNAs translationally controlled by Xrn1. **a**, **b** Box-plot depicting the mean length (**a**) and the mean PARS score (**b**) of the 5ʹUTRs, CDSs and 3ʹUTRs for the three subgroups studied and the control group (gray). For boxplots, box boundaries represent the 1st and 3rd quartile of the distribution, while the center line represents the 2nd quartile (median). Whiskers indicate either the most extreme values or extend to 1.5 times the interquartile range starting from the respective box boundary. Black dots indicate outliers (default R parameters). Statistical significance was calculated using a Wilcoxon-test (*p*-values available in Supplementary Data 4). **c** Metagene analysis of the PARS scores. Vertical dashed line corresponds to the translation initiation site (TIS). The *x*-axis represents the nucleotide position relative to the TIS. **d** Metagene analysis of the average mRNA-normalized RPF coverage along the CDS for activated (red, mRNA=), repressed (green, mRNA = and orange, mRNA ↓), and not affected mRNAs (gray). A light red line corresponds to untreated cells, whereas turquoise corresponds to treatment with auxin for 30 min

Next, we investigated whether mRNAs regulated by Xrn1 share common physical properties. First, we calculated the average length of the 5ʹUTRs, CDSs and 3ʹUTRs for the three groups and compared them to all genes that were not significantly altered (Fig. 6a and Supplementary Data 4a–d). Xrn1-activated mRNAs had significantly longer 5ʹUTRs (80 nt) and CDS (1555.5 nt) when compared to transcripts not significantly altered (52 and 1113 nt). In contrast, Xrn1-repressed mRNAs had shorter 5ʹUTRs (mRNA buffered: 44; mRNA decreased: 35.5 nt) and CDS (mRNA buffered: 753; mRNA decreased: 600 nt) compared to not significantly altered transcripts, whereas only repressed genes with buffered mRNA levels showed an increased 3ʹUTR length (128 nt) compared to background genes (105 nt). Interestingly, as for the cellular Xrn1-activated mRNAs, BMV RNA2 contains long 5ʹUTRs and CDS (92 nt and 2468 nt). Second, as BMV RNA2 5ʹUTR contains highly structured sequences, we examined whether this feature also characterizes the cellular Xrn1-activated mRNAs and extended this analysis to the CDS. We used previously published datasets of genome-wide RNA secondary structure obtained by PARS (Parallel Analysis of RNA structure)^26^ to analyze the RNA structure profile in the 5ʹUTRs, CDSs and 3ʹUTRs. PARS scores are based on deep-sequencing of RNA fragments obtained by RNA digestion with enzymes that exhibit structural preferences. While there were no substantial differences in the 3ʹUTR, all groups of transcripts regulated by Xrn1 had a significantly higher PARS score in the CDS (0.32–0.35) compared to transcripts not significantly affected by Xrn1 (0.25) (Fig. 6b). Importantly, only activated transcripts had a higher structured 5ʹUTR (0.16) compared to transcripts not significantly affected (0.06). Repressed genes, whose mRNA levels decreased due to depletion of Xrn1, exhibited the lowest structure of the 5ʹ-UTRs (0.03). These differences are more visible when plotting the PARS score distribution (Fig. 6c). Note, in particular, that the PARS score drops around the translation initiation sites (TIS) (Fig. 6c). This relatively unstructured region has probably evolved to permit an easy access to the ribosome at the canonical translation initiation site, and may contribute to the recognition of true start sites^26,27^. Interestingly, Xrn1-activated mRNAs have a substantially higher PARS score at the TIS and their upstream regions, suggesting that one of the Xrn1 functions is to override the structural barrier at the TIS. Notably, we found that long 5ʹUTRs and CDSs and highly structured 5ʹUTRs with unfavorable contexts for translation initiation are indeed common features for mRNAs encoding membrane proteins (as defined in ref. ^24^). Remarkably, those mRNAs identified to depend on Xrn1 for translation show the highest PARS scores (Supplementary Fig. 10).

To further understand the role of Xrn1 in translational regulation, we questioned whether the changes observed in ribosome occupancy in activated transcripts were due to changes in initiation and/or elongation. Ribosome-profiling can detect defects in specific translation steps because discrete ribosome pausing increases the likelihood of capturing footprints in the pausing site by deep-sequencing. Therefore, defects in elongation caused by ribosome stalling result in a peak in ribosome density and an accumulation of ribosomes upstream of the stalling site. This would be visible as a change in slope in a metagene analysis, with the 5ʹ end showing an increased footprint density and the 3ʹ end showing a decrease. In contrast, differences in translation initiation result in a shift of ribosome occupancy along the entire CDS. To distinguish between these two possibilities, we examined the relative RPF distribution along the CDS of the three mRNA groups defined previously and compared them to genes that did not show any significant alterations (Fig. 6d). In agreement with a role of Xrn1 in translation initiation, activated genes exhibited a general reduction of footprint density while repressed genes exhibited a general increase. Collectively, we conclude that long 5ʹUTRs and CDSs and highly structured 5ʹUTRs are common features of both BMV RNA2 and cellular transcripts that depend on Xrn1 for translation. This suggests a common role for Xrn1 in regulating translation of cellular mRNAs and BMV RNA2, likely at the translation initiation step.

### The distinct functions of Xrn1 in gene expression are linked

**Fig. 7:**
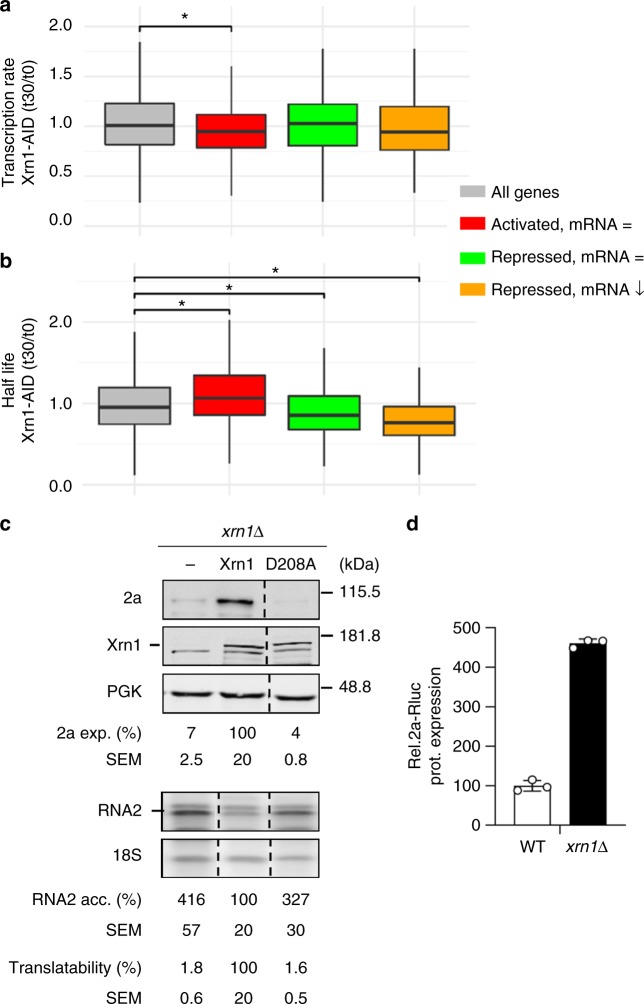
Xrn1 functions in mRNA transcription, decay, and translation are linked. **a** Transcription rates and **b** half-lives of the studied mRNA groups. Statistical significance was calculated using a Wilcoxon-test (*represents *p*-value < 0.001) The results represent an average of *n* = 3 biological replicates. For boxplots, box boundaries represent the 1st and 3rd quartile of the distribution, while the center line represents the 2nd quartile (median). Whiskers indicate either the most extreme values or extend to 1.5 times the interquartile range starting from the respective box boundary. **c** Xrn1 D208A effect on BMV RNA2 translation. **d** When introduced directly into the cytosol by electroporation, RNA2 does not depend on Xrn1 for translation. RNA2-Rluc mRNA and a control mRNA expressing Fluc were directly electroporated into the cytosol. 2a-Rluc protein values were normalized by those of Fluc and represented in comparison to the WT. Quality controls for RNA integrity and capping were performed (Supplementary Fig. 12). The results represent an average of *n* = 3 biological replicates ± SEM. Open circles indicate the individual dot plots. Source data are provided as a Source Data file

To examine whether the function of Xrn1 in translation is linked to its known roles in transcription and decay, we determined the effect of Xrn1 on the transcription and decay of those mRNAs that are translationally activated by Xrn1. To obtain a whole-genome view of transcription rates (TRs), we performed genomic run-on experiments (GRO)^28^ in the Xrn1-AID degron system 30 min after auxin addition. In parallel with GRO analysis, we determined the mRNA steady-state levels, which allowed us to calculate mRNA half-lives^2^. Reassuringly, we found that depletion of Xrn1 for 30 min had a similar effect on transcription rates to that previously observed in *xrn1*Δ strain^1,2^. The GO categories (Supplementary Data 5) enriched in genes transcriptionally activated (ribosome biogenesis, translation) or repressed (mitochondria, respiration) by Xrn1 were similar to those observed when Xrn1 is permanently depleted although quantitatively lower, suggesting that permanent depletion of Xrn1 intensifies this phenotype. Likewise, the distinction between the Xrn1 Synthegradon group, which is highly responsive to *XRN1* disruption, and the anti-Synthegradon group, which contains the least responsive genes^2^, was clearly visible upon Xrn1-AID depletion (Supplementary Fig. 11). Importantly, within the groups of mRNAs translationally regulated by Xrn1, the translationally activated one showed significantly decreased transcription rates and increased half-lives upon Xrn1 depletion (Fig. 7a, b = mRNA, red). Thus, Xrn1 stimulates mRNA synthesis, translation, and decay of this group of mRNAs.

Next, we investigated whether these three Xrn1-driven functions are linked. If this is the case, defects in one function should affect the others. To address this key issue, we made use of the catalytically inactive *xrn1*^D208A^ mutant. This mutant binds decapped mRNAs but is unable to degrade them and becomes trapped in an RNA-bound state^29,30^. Consequently, the nuclear import of Xrn1, and other mRNA decay factors whose import depend on Xrn1, is impaired^1,31^. Replacing *XRN1* at its genomic locus by *XRN1*^D208A^ results in transcription and decay defects comparable to those in *xrn1∆* cells^1^. We reasoned that if the function of Xrn1 in translation, transcription and decay are linked, *xrn1*^D208A^ mutation should also impair Xrn1-dependent translation. Ribosome-profiling analyses of the *xrn1*^D208A^ mutant identified a group of cellular mRNAs that depend on Xrn1 for translation. These mRNAs were enriched for cellular functions related to protein glycosylation and membrane localization (Supplementary Data 6) and significantly overlapped with those identified with the Xrn1-AID system (*p*-value = 1.4e^−29^, Hypergeometric test). Moreover, *xrn1*^D208A^ mutation inhibited BMV RNA2 translation like *XRN1* deletion (Fig. 7c). Similarly, a catalytically inactive version of Rat1ΔNLS-XC (Rat1ΔNLS-XC-D235A) inhibited RNA2 translation (Supplementary Fig. 8a, b). To obtain further insights into the linkage between the three functions of Xrn1, we uncoupled RNA2 translation from transcription. We reasoned that if Xrn1-dependent translation requires a previous function of Xrn1 in transcription, introducing RNA2 directly into the cytosol would result in an Xrn1-independent translation of RNA2. To test this, we electroporated in vitro transcribed RNA2-Rluc into WT and *xrn1Δ* cells together with an mRNA control expressing Firefly-luc for normalization (Fig. 7d). Remarkably, in contrast to the inhibition of 2a protein expression observed in *xrn1Δ* when RNA2 was expressed from a plasmid (Fig. 1a), deletion of *XRN1* did not inhibit 2a protein expression (Fig. 7d). Instead, we observed an increase in the expression of 2a protein consistent with an extended RNA2 half-life (Fig. 4a), indicating that the linkage between transcription and post-transcriptional stages was broken by circumventing transcription and introducing mRNAs by electroporation. Altogether, we conclude that the functions of Xrn1 in synthesis, translation, and decay of the translationally activated mRNAs are linked.

## Discussion

In this study, we reveal an unanticipated role for the major 5ʹ–3ʹ exonuclease Xrn1 in both activating translation of mRNAs encoding membrane proteins and in directing these mRNAs to the ER, their translation site. Remarkably, for this group of mRNAs Xrn1 activates translation, transcription and decay, and these functions are linked. Our results uncover a crosstalk between the three major processes of gene expression coordinated by Xrn1 to express membrane proteins.

A number of observations are consistent with a specific role of Xrn1 in translation initiation. First, transient depletion of Xrn1 has an immediate inhibitory effect on RNA2 translation. Consequently, a selection of adaptive mutations that may account for the observed translation effects seems unlikely (Fig. 3d, e). Second, Rat1ΔNLS, the nuclear paralog of Xrn1 retained in the cytoplasm due to the deletion of its NLS, fully rescues RNA2 stability (Fig. 4a), but not RNA2 translation (Fig. 4b). These data argue against the possibility that changes in mRNA abundance driven by Xrn1 depletion are responsible for the observed translation defects. It has been reported that Xrn1 depletion causes changes in the formation of RNA duplexes that result from transcription of convergent genes and affect their protein expression levels^32^. Our ribosome-profiling data show, however, no enrichment for convergent genes. Third, polysome-profiling analyses demonstrate that, upon Xrn1 depletion, BMV RNA2 shifts from polysomes to monosomes (Fig. 2b) and ribosome-profiling analyses show a decrease in the footprint density along the CDSs of cellular mRNAs that are translationally activated by Xrn1 (Fig. 6d). These features are consistent with effects in translation initiation. The co-fractionation of Xrn1 with 40s ribosomal subunits would point to a role of Xrn1 in early translation initiation events (Fig. 2c). Fourth, Xrn1 but not Rat1ΔNLS interacts genetically^33^, physically, and functionally (this study) with the translation initiation factor eIF4G. This was shown by immunoprecipitation analyses (Fig. 4c, d) and gain-of-function studies in which the C-terminal domain of Xrn1, the only domain not present in Rat1, was fused to Rat1ΔNLS (Fig. 4d). This domain is highly disordered and includes short linear motifs (SLiMs), features typical of protein sequences with capacity to bind multiple interacting partners^34^. Such motifs also characterize the Xrn1 homologs in *Homo sapiens* and *Drosophila melanogaster*^6^. Last, the mRNAs whose translation is activated by Xrn1 have an unfavorable translation initiation context. They contain long and highly structured 5ʹUTRs as demonstrated by PARS scoring for host mRNAs and by functional studies for RNA2^26,35^. Typically, PARS scores drop immediately upstream of the translation initiation site to favour translation initiation. In contrast, the mRNAs that are translationally activated by Xrn1 exhibit an unusual high PARS score in this region, suggesting that one function of Xrn1 is to overcome this impediment. Reassuringly, replacement of the highly structured 5ʹUTR in RNA2 resulted in a 35-fold increase of translatability in WT cells and a 13-fold decrease in Xrn1-dependence (Fig. 1b).

Xrn1 coordinates a linkage between transcription, translation, and decay for a specific group of mRNAs enriched in transcripts encoding membrane proteins. We surmise that this linkage has evolved to control proper gene expression of membrane proteins. These proteins contain hydrophobic domains with strong tendencies to aggregate. Consequently, their expression levels and localization must be finely tuned to avoid aggregations that might be toxic. Indeed, mRNAs related to vacuole transport in yeast and the endomembrane system in *Arabidopsis thaliana* are among the most frequently co-translationally degraded by Xrn1^8,36^. This feedback mechanism would ensure that Xrn1-dependent mRNAs are efficiently translated only when decay is working properly. The decision to express a gene, or a family of genes, is obtained by crosstalks between all major stages of the mRNA lifecycle. For example, it is counterproductive to transcribe a gene if the translation apparatus is not capable of translating it. One mechanism to permit these crosstalks is by factors that function in all these stages. Xrn1 seems to carry this function. Another example is Rpb4/7, that also acts in transcription, mRNA export, translation, and decay^37^. Interestingly, *XRN1* and *RPB7* interact genetically^38^, raising the possibility that they function in a coordinated manner. One advantage for the cell of using factors that function in all stages of the mRNA lifecycle is the ability to regulate the synthesis and functionality of mRNAs by regulating these factors.

In our study, we also identified two subsets of mRNAs translationally repressed by Xrn1. One of them is enriched for functions related to proteasomal degradation and protein folding (Fig. 5c). This hints to a crosstalk of mRNA biogenesis and turnover with protein degradation. The other subset of mRNAs is enriched for functions related to translation and ribosomes. Interestingly, these mRNAs are the most dependent on Xrn1 for transcription and decay at optimal growth conditions^2^. This would allow a precise control of the global translation, the most energy consuming cellular process. Although the mechanistic details of Xrn1 function in translation repression are unclear, our data indicate that it is context dependent (Fig. 6a–c) and, in the case of repressed mRNAs, not linked to transcription and decay activation (Fig. 7a, b).

Based on our observations, we propose a model wherein Xrn1 promotes translation initiation of mRNAs with unfavorable translation initiation contexts by interacting with eIF4G and stabilizing the interaction of the scanning 40s subunit with the mRNA. As eIF4G shuttles to the nucleus, where it is proposed to function in splicing^39^, an interesting possibility is that the two shuttling proteins interact in the nucleus or even shuttle together. By promoting translation, Xrn1 will then favour co-translational targeting of mRNAs to the ER. Alternatively, Xrn1 may assist mRNA localization at the ER by a translation-independent mechanism. Once at the ER and after several rounds of translation, the mRNAs would then be co-translationally degraded by Xrn1.

We propose that the Xrn1-mediated crosstalk between transcription, translation, and decay involves the decaysome complex. The decapping activators Lsm1–7, Pat1, and Dhh1 shuttle to the nucleus in an Xrn1-dependent manner, associate to chromatin and stimulate transcription^1^. Likewise, these decapping activators are required for the function of Xrn1 in translation. Lsm1–7, Pat1, and Dhh1 are required for BMV RNA2 translation and the DEAD-box RNA helicase Dhh1 activates translation of cellular mRNAs^13^ that significantly overlap with those translationally activated by Xrn1 (*p*-value = 1 × 10^−5^, Hypergeometric test). However, our data support that each factor carries a distinct non-overlapping function that complements each other. For example, Lsm1–7/Pat1-requirements for BMV RNA translation are related to sequences within the 3ʹUTR^40,41^, Dhh1-requirement to sequences within the 5ʹ and 3ʹ UTRs and CDS^13^ and Xrn1-requirement primarily to sequences within the 5ʹUTR (this study).

Collectively, our data provide mechanistic details of the function of Xrn1 in translation activation and uncover an Xrn1-mediated crosstalk between the three major stages in gene expression. A key area for future research will be in elucidating how the transitions between the subsequent Xrn1 functions are regulated and responsive to the environment.

## Methods

### Yeast cultures

Yeast cells were grown in synthetic complete medium (SC) at 30 °C. Galactose (2%) was used as carbon source and it also served as inductor for *GAL1*-directed viral RNA expression. After transformation, three colonies for each condition were selected and streaked on a selective media plate. Cells were grown over-day in selective liquid media and diluted to grow overnight. Next day, they were diluted and grown until the doubling time between triplicates was similar and an OD_600_ of ~0.6 was reached. Yeast strains and plasmids are listed in Supplementary Data 7.

### Cloning of Xrn1 derivative strains

Xrn1-AID strain was generated by amplification of the cassette present in BYP7427 plasmid with Xrn1-specific primers. The PCR product was purified and transformed in BY25598 strain and plated in minimal media with corresponding selection markers (−His, +Ade, +Leu, +Tryp). Positive colonies were selected by checking the correct integration of the AID cassette in Xrn1 and were subsequently sequenced.

Xrn1 D208A strain was generated by the following strategy. First, *XRN1* gene was amplified and cloned in pSZ229 using Fw-Xrn1–250-AgeI and Rv-Xrn1 + 110-AcI primers. Second, this plasmid was transformed in BY4741 *xrn1∆* strain and plated in +G418 +His selective plates for homologous recombination to occur. Third, colonies were tested by PCR to ensure recombination had occurred in the right locus and subsequently sequenced. All primers and strains are listed in Supplementary Data 7 and Supplementary Data 8.

### Cloning of Xrn1 derivative plasmids

The plasmids generated for this study were cloned following conventional molecular cloning strategies. In order to be able to select for all plasmids used in this study, we changed the selection markers of plasmids pAJ37 and pAJ228^19^ by URA3 marker, and we named the plasmids pBBM1 and pBBM3, respectively. Xrn1 D208A mutant plasmid (pBBM2) was generated using the KOD Hot Start DNA polymerase from Millipore.

pLCM2 was generated by inserting one single copy of a FLAG-tag (GATTACAAGGATGACGACGATAAG) in the 3ʹ end of pBBM2. To increase our signal, we inserted two more copies in pLCM2, generating a 3xFLAG (GATTACAAGGATGACGACGATAAGGACTATAAGGACGATGACGACAAAGATTATAAAGACGACGATGACAAG) Rat1∆NLS plasmid (pLCM6). All the experiments done along this study have been performed with pLCM6 and we refer to it as Rat1∆NLS-FLAG plasmid. pLCM7 was generated by taking out the 3xFLAG from pLCM6 and introducing it in the 3ʹ end of pBBM1 plasmid.

Rat1∆NLS-XC-FLAG chimera (pLCM9) was generated by fusing the N-terminal domain (1–884 aminoacids) of Xrn1 in pBBM3 to the C-terminal domain (733–1528 aminoacids) of Rat1∆NLS in pLCM7. Also, we depleted a loop in the CDS of Rat1∆NLS (∆24–42 aminoacids) because it was hampering the interaction between the N-terminal and the C-terminal domains of the chimera.

Rat1∆NLS-XC-D235A-FLAG mutant plasmid (pLCM22) was generated using the KOD Hot Start DNA polymerase from Millipore.

All the plasmids and primer sequences used in this study are listed in Supplementary Data 7 and 8.

### BMV RNA translation assay

To evaluate BMV protein translation, yeast cells were transformed with the corresponding plasmids and grown as specified in the previous section. Two OD units (Optical Density units) were harvested for protein extraction and three OD units for total RNA extraction.

Total protein was extracted from equivalent number of cells and loaded on an sodium dodecyl sulfate polyacrylamide gel electrophoresis (SDS-PAGE) gel to be separated according to their molecular weight. Next, samples were immunoblotted into a nitrocellulose membrane for 90 min at 100 V on ice, as previously described^42^. Antibodies against 2a protein^15^, GFP, PGK (Molecular Probes), and Xrn1 (gift from Arlen Johnson) were used (Supplementary Data 9). Detection of 2a protein was done with FUJIFILM Luminiscent Image Analyzer LAS-1000. For the rest of proteins, the infrared imaging system Odyssey (LI-COR Biosciences) was used.

Total RNA from yeast cells was isolated by a hot-phenol method, concentration was measured with a Nanodrop device and 3 µg of total RNA were loaded on formaldehyde denaturing agarose gels for subsequent northern blot analysis^43^. MAXIscript in vitro transcription kit (Ambion) was used to generate probes that specifically detect RNA2, GFP RNA, and 18S RNA. The generation of these probes by in vitro transcription was based on previously described plasmids^42,44,45^. Northern blots were developed by exposure to PhosphorImager screens and imaging on a Typhoon 8600 (Amersham). Quantification was carried out by measuring band intensity using the ImageQuant software (Molecular Dynamics).

### Protein turnover assay

Yeast cells transformed with a plasmid encoding for RNA2-Rluc reporter (pJJ-16) were grown as described in Yeast Cultures section. When they reached an OD_600_ of 0.5, protein synthesis was stopped with 0.5 mg/ml cycloheximide. Renilla luciferase activity assay (Dual-Glo®, Promega) was used following the protocol provided by the manufacturer. Samples were collected at different intervals during 3 h by directly transferring 10 µl of culture to 100 µl of 1x Passive Lysis Buffer. Only 10 µl of the lysate were used (the rest was stored at −80 °C) and 200 µl of LARII-StopGlo solution (1:1) was subsequently added. FB12 Luminometer was employed to read Luciferase activity, with 5 s of equilibration time and 5 s of measurement time. The values obtained were corrected by the corresponding OD_600_ and were represented relatively to the first time-point (*t* = 0). This protocol was adapted from ref. ^46^.

### RNA ligase-mediated (RLM)-Rapid amplification of cDNA ends (RACE)

To characterize the 5′ terminus of the BMV RNA2, total RNA obtained by phenol extraction was used to perform 5ʹ RNA Ligase-mediated (RLM) rapid amplification of complementary DNAs (cDNAs) ends using a FirstChoice RLM-RACE Kit (Thermo Fisher Scientific) following manufacturer’s instructions. For the outer 5ʹ RLM-RACE PCR, 5ʹ RACE outer primer and gene-specific primer (5ʹ-CATTTGTTGGACGGTGTCGCAA-3ʹ) were used. One thousand dilution of the above-mentioned PCR was used to amplify a nested PCR fragment with a 5ʹ RACE inner primer and a gene-specific primer (5ʹCTCCTATCTCCAAGGGCGCTAT 3ʹ).

### Polysome profiling

Cultures were grown from OD_600_ = 0.02 to an OD_600_ = 0.5 in YPD media (Formedium) at 30 °C. In order to stabilize elongating ribosomes, cells were treated with cycloheximide (CHX, 100 µg/ml final concentration) during 1 min with manual shaking at room temperature. Cells were quickly harvested with a vacuum filtration system, scraped out of the filter and immediately frozen in liquid nitrogen with 500 μl of lysis buffer (20 mM Tris-HCl (pH = 7.5), 100 mM NaCl, 5 mM MgCl_2_, 1% Triton X100, 0.5 mM DTT, 100 μg/ml CHX). Cells were lysed with the Freezer/Mill (SPEX SamplePrep) with two cycles of 2 min at 5 cps with a 2 min cooling-down step in between. Cell lysates were thawed at 30 °C for 1 min and centrifuged at 3000 × *g* and 4 °C for 3 min. The soluble fraction was transferred to new tubes and centrifuged at 10,000 × *g* and 4 °C for 5 min. After quantification, aliquots of 12 UA_260_ were made and stored at −80 °C. Linear gradients of 10–50% sucrose were prepared in 50 mM Tris-HCl (pH = 7.5), 50 mM NH_4_Cl, 12 mM MgCl_2_, 0.5 mM DTT, 100 μg/ml CHX. The Gradient Master (Biocomp) was used for making the gradients in 14 × 89 mm polyallomer tubes (331372, Beckman Coulter). One aliquot of 12 UA_260_ was loaded on each gradient and centrifuged in a Beckman SW41 rotor at 209,490 × *g* and 4 °C for 3 h. Gradients were fractionated with fraction collector Model 2128 (Biorad). These fractions were used for hot-phenol RNA extraction or TCA protein precipitation and analyzed by northern blot or western blot, respectively.

### Co-immunoprecipitation

Yeast cells carrying genomic GFP-tag fusions of either eIF4G, eIF4E, or eIF4A were transformed with Xrn1, Rat1ΔNLS, and Rat1ΔNLS-XC FLAG-tagged plasmids and were grown (400 ml culture) in exponential phase until an OD_600_~0.6 was reached. They were harvested by centrifugation (5 min, 1811 × g, 20 °C) and lysed by vortexing with glass beads (four cycles of 30 s and four cycles of 1 min) in lysis buffer (50 mM Tris-HCl (pH = 7.5), 150 mM NaCl, 0.1% NP-40, 1 mM EDTA and protease inhibitors). After recovering the soluble fraction, total protein amount was measured by Pierce TM BCA Protein Assay Kit (ThermoFisher). As a control for the input sample, 100 μg of total protein were kept. For the immunoprecipitation, 3 mg of total protein were used. Samples were measured in the Nanodrop and 1.12 U RNaseI/10 AU_260_ were added in the RNase-treated samples. These were incubated for 1 h at 22 °C with inversion mixing every 10 min. In the meantime, untreated samples were kept at 4 °C. +/– RNase-treated protein extracts were then incubated with 15 µl of GFP-trap_A beads (Chromotek) for 1 h at 4 °C shaking in a rotating mixer. Three washes with 500 µl of wash buffer (10 mM Tris-HCl (pH = 7.5), 200 mM NaCl, 0.5 mM EDTA, 1 mM PMSF, Protease inhibitor Cocktail) were performed and beads were pelleted in-between by centrifugation (2500 × *g*, 2 min, 4 °C). Beads were resuspended in 20 µl of wash buffer and 10 µl of 3x loading dye were added. Samples were eluted from the beads by boiling at 95 °C for 5 min. The antibodies used are listed in Supplementary Data 9.

### RNA stability assay

Yeast cells transformed with the desired plasmid were grown in selective SC media with 2% galactose, as described in the Yeast Cultures section. Cultures of 50 ml were inoculated and were grown until they reached exponential growth and an OD_600_ of 0.7. Three OD units were harvested by centrifugation (1500 × g, 4 °C, 3 min) and frozen directly in liquid nitrogen. The rest of the yeast culture was centrifuged simultaneously and the media was exchanged with new pre-warmed media containing 2% glucose, in order to shut-off the transcription of BMV RNA2. Samples of three OD units were harvested at different time-points (15, 25, and 60 min) and frozen. Total RNA extraction and northern blot analysis were performed.

### Translation assay upon Xrn1 auxin-induced degradation

A yeast strain with an integrated TIR1 was used for the generation of a genomic fusion of Xrn1 to an auxin-induced degron (AID). Together with TIR1, this fusion enabled the quick degradation of Xrn1 protein upon addition of auxin, taking advantage of a protein degradation pathway in plants^18^.

Yeast cells transformed with BMV RNA2-Rluc plasmid were grown in SC media with 2% raffinose until they reached exponential phase and an OD_600_~0.5 in 50 ml cultures. Galactose (2%) and auxin (500 µM) were added to induce BMV RNA2-Rluc expression and deplete Xrn1, respectively. Samples were taken at different time-points for OD measurement, Luciferase activity assay and total RNA extraction. BMV RNA2-Rluc was quantified by reverse transcription quantitative PCR using TaqMan probes and the qScript XLT One-Step RT-qPCR ToughMix (Quanta Biosciences).

### Ribosome profiling

Ribosme profiling experiments were performed essentially as described in ref. ^47^. Yeast cells were grown, harvested, and lysed as described in the Polysome Profiling section. In the case of ribosome-profiling, 10 OD260 units of lysates were treated with 112.5 U of RNaseI (Ambion) for 60 min at 22 °C and 1400 rpm in the Thermomixer. RNaseI activity was stopped by addition of 100 U of SUPERaseIn (Ambion) and digested extracts were loaded in 7–47% sucrose gradients. The preparation of gradients followed the same protocol as in polysome profiling, but in this case SUPERaseIn was added to the gradients as well (10 U/ml) Ultracentrifugation was performed for 3 h at 209,627.4 × *g* and 4 °C in a TH-641 rotor (Thermo Scientific). The fractionation of gradients was performed with a Density Gradient Fractionating System (Brandel) at a rate of 0.75 ml/min. Monosomal fractions corresponding to digested polysomes were collected, SDS to 1% was added to stop any possible RNase activity and samples were flash-frozen in liquid nitrogen and stored at −80 °C. RNA was isolated from monosomal fractions using the hot acid-phenol method. Ribosome-protected fragments (RPFs) were isolated by running 15% polyacrylamide, 8 M urea, 1X TBE gels and isolating RNA fragments of 28–32 nucleotides (nt). For RNAseq, 150 µl of the same lysate were used for total RNA extraction with the hot acid-phenol protocol and subsequent TURBO DNase treatment (Ambion). Total RNA was quantified and 100 μg were used for two rounds of purification with the Poly(A)Purist MAG kit (Ambion). Next, the purified mRNA was fragmented by alkaline hydrolysis in 50 mM sodium bicarbonate (pH = 9.2) and 1 mM EDTA for 20 min at 95 °C. The RNA was purified by ethanol precipitation and fragments of 50–80 nt were selected on a 15% polyacrylamide, 8 M urea, 1 × TBE gel. The ligation of the 3ʹ-adapter was performed for 4 h at 22 °C with 200,000 U of T4 RNA ligase 2 (truncated, NEB), 25% PEG 8000 and 10 U of SUPERase In.

### Genomic run-on

Genomic Run-On (GRO) was done in biological triplicates essentially following the protocol described in ref. ^48^. We used the same number (5 × 10^8^) exponentially growing yeast cells (OD_600 = _0.5) for each run-on reaction. Another aliquot of the same cells was used directly for RNA extraction, which subsequently was used for cDNA synthesis using ^33^P-dCTP. GRO samples provided nascent transcription rates (nTR) for every yeast gene. Then, they were corrected by average cell volume (the median of the population measured by a Coulter Counter device) for times 0 and 30 min after auxin addition to obtain mRNA synthesis rates (SR). Whole RNA polymerase II transcriptome SR was obtained by summing up all individual genes SR data. Transcriptome data (mRNA levels, RA) were obtained from the hybridization of labeled cDNA onto nylon filters. Total mRNA concentration in yeast cells was determined by quantifying polyA + in total RNA samples by oligo-dT hybridization of a dot-blot following the protocol described^49^ and dividing by average cell volume. mRNA half-lives (HL) in arbitrary units for every mRNA were obtained by dividing individual RA values by SR ones^1^.

### Ribosome-profiling analysis

Ribosome-profiling reads were aligned to the sacCer3 transcriptome (SGD annotation) with bowtie^50^ using the following settings: “-S -t -p 30 -n 1 -m 1 -l 25 –norc.” The transcriptome consisted of all CDS flanked by 18 nt of genomic sequence on either side (representing the UTR). Reads mapping to the first 63 nucleotides (18 nt 5ʹ UTR + 15 codons) were discarded to remove cycloheximide-induced artefacts. After codon assignment and quantification (analogous to ref. ^13^), raw per gene counts were supplied to the Riborex R-package v1.2.3^23^ to identify significant differences of translational regulation upon Xrn1 knock-down (minMeanCount = 0; FDR < 0.05). As Riborex only provides a fold change estimate for the interaction between condition (t0 vs. t30) and technique (mRNA vs. RPF), all significant genes were grouped according to their behavior relative to the diagonal (=no change of translational control) by obtaining the corresponding DESeq2^51^ moderated log_2_ fold changes for mRNA and RPF samples separately (as displayed in Fig. 5b). To simplify subsequent analyses, we considered genes whose mRNA log_2_ fold change was smaller than ± 0.433 (analogous to ref. ^13^) to have buffered/stable levels of mRNA.

Gene ontology enrichment analysis for the resulting groups was performed using gProfileR^52^ with settings “correction_method = ‘‘fdr’’ and “hier_filtering = ‘‘moderate’”. For visualization, we used REViGO to define redundant GO terms (http://revigo.irb.hr/) and only considered the top 5 non-dispensable terms.

For the metagene coverage analysis, per codon RPF counts were normalized by gene length and library size (observed/expected) before being averaged per condition. The averaged normalized footprints were then corrected by the corresponding mRNA fold change relative to t0. Visualization was done using the ggplot2 R-package^53^.

### Analysis of PARS scores

Nucleotide resolution PARS scores for yeast RNAs were obtained from GitHub (https://github.com/abelew/prfdb/tree/master/pars/sce_Score.tab) and further processed using the statistical programming language R. For a high resolution analysis of the region surrounding the translation initiation site (TIS) we extracted up to 100 nt of the 5ʹ UTR (if available) as well as the first 300 nt of the coding sequence (CDS) and plotted the mean (na.rm = TRUE) PARS profiles for all the groups studied. PARS scores were also averaged across the 5ʹUTR, CDS and 3ʹUTR in order to compare increased or decreased RNA structure in certain RNA regions between the different groups. Unless specified otherwise, a Wilcoxon-Mann–Whitney test was used to detect significant differences in all analyses.

### Structure modeling of the chimera Rat1∆NLS-XC

The sequences of Rat1∆NLS and Xrn1 were aligned with the sequences of template structures of *S. pombe* and *K. lactis* taken from the Protein Data Bank (PDB)^54^, with codes 3FQD and 3PIF, respectively. We superposed both structures with MATCHMAKER, using CHIMERA^55^, and detected that the structure of Rat1 deviates from Xrn1 at the position of Arg 653 in chain A of 3FQD, which corresponds to position 884 in the sequence of Rat1, and this was selected for merging both the sequences.

The N-terminal domains of both structures are very similar. However, we detected a protruding loop on the structure of Rat1 that collided with the C-terminal domain of Xrn1 (between Ile19 and Gln25 positions in chain A of 3PIF, corresponding to positions Val21 and Pro45 in chain A of 3FQD, aligned with the sequence LEEQPQIVDGVIL of Rat1∆NLS, see Supplementary Fig. 7). This loop was removed from the sequence to construct the chimera sequence and test its foldability. The chimera sequence merged Rat1∆NLS and Xrn1, which was aligned with the sequences of the templates (3FQD and 3PIF) using Clustalw^56^. The structure was modeled with MODELLER^57^ and optimized with Rosetta^58^.

### Cellular fractionation for mRNA recruitment analysis

Xrn1-AID strain was grown in YPD with 2% glucose at 30 °C until the doubling time between triplicates was similar and an OD600 of ~0.5 was reached. Ten ODs of the culture were harvested (=WT). In the remaining culture the degradation of Xrn1 was induced for 30 min by adding auxin to a final concentration of 0.5 mM. After 30 min, cells (10 ODs) were harvested by centrifugation. To analyze the RNA distribution between cytoplasm and membrane fractions 240 µl of lysis buffer (50 mM MOPS, 275 mM potassium glutamate, 5 mM Mg Acetate and freshly added 100 µg/ml CHX, 1 mM DTT, 20 U/ml Superasein and Roche protease inhibitor) were added to ten ODs of frozen yeast pellet. Cells were lysed cryogenically by mixer milling in a Retsch MM400 at 30 Hz 2 min (four times). The lysate was thawed at 25 °C and centrifuged 10 min at 13,400 × *g*. The supernatant, corresponding to the cytoplasm, was transferred to a new eppendorf tube and triton was added to 0.1%. The pellet was resuspended in lysis buffer containing 1% triton and homogenized in a dounce homogenizer. After centrifugation (10 min 13,400 × *g*) the supernatant contains the membrane fraction. RNA extraction was carried out using the hot-phenol method. mRNA distribution between cytosol and membrane fraction was analyzed by reverse transcription quantitative (q)PCR using TaqMan probes and qScript XLT One-Step RT-qPCR Though Mix from Quanta Biosciences. Twelve nanograms of total RNA were loaded and amplified using specific primers. The list of primers is available in Supplementary Data 8.

### Electroporation of in vitro transcribed RNA2-Rluc

The protocol used is an adaptation of the one previously described^59^. Reporter RNAs (RNA2-Rluc and pLucA) were in vitro transcribed from a plasmid using the MAXIscript T7 In Vitro Transcription Kit (Ambion) and the MAXIscript T3 In Vitro Transcription Kit (Ambion), respectively. The transcripts were subsequently capped with ScriptCap™ m^7^G Capping System (CELLSCRIPT). Their integrity was evaluated with a denaturing formaldehyde gel. After quantification with a Nanodrop device, RNAs were aliquoted and stored at −80 °C.

Yeast cells (wt and *xrn1∆*) were treated with lyticase to remove the cell wall and incubated with Sorbitol 1 M. Next, the spheroplasts were incubated with YAPD-Sorbitol 1 M for 90 min at 30 °C to allow cell recovery. Finally, cells were pelleted, resuspended with Sorbitol 1 M, and kept on ice. Electroporation cuvettes (0.2 cm electrode gap, BioRad, Hercules) were kept on ice and RNA2-Rluc and pLucA RNA were added (8 and 0.5 µg, respectively). Yeast spheroplasts were pipeted (180 µl) into the cuvette and pulsed immediately (Bio_rad gene pulser II: 800 V, 25 µFaraday and 1000 Ω, 20–25 ms). Spheroplasts were transferred to ice-cold 2 ml tubes and placed at 30 °C with gentle swirling. After 30 min, cells were pelleted (3 min, 1500 × g), the supernatant was discarded and the pellets frozen immediately in liquid nitrogen (stored at −80 °C). For measurement, cells were resuspended in 50 µl of 1X reporter lysis buffer (Renilla Luciferase activity assay, Dual-Glo®, Promega) and vortexed vigorously for 30 s. Cell lysate (20 µl) and 50 µl of luciferase assay substrate were mixed and measured immediately.

### Statistical information

All the statistical information is detailed throughout the Figure legends and the methods section.

### Reporting summary

Further information on experimental design is available in the Nature Research Reporting Summary linked to this article.

## Supplementary information

Supplementary Information

Peer Review

Reporting Summary

Description of Additional Supplementary Files

Supplementary Data 1

Supplementary Data 2

Supplementary Data 3

Supplementary Data 4

Supplementary Data 5

Supplementary Data 6

Supplementary Data 7

Supplementary Data 8

Supplementary Data 9

Source data

## Data Availability

A reporting summary for this Article is available as a Supplementary Information file. Ribosome Profiling and Genomic Run-On (GRO) raw data are available under accession GSE109734 and GSE123326 at Gene Expression Omnibus (GEO). The source data underlying Figs. 1–5, 7, and Supplementary Figs. 1–2, 3–8, 12 are provided as a Source Data file.
