# Supplementary material for: The exonuclease Xrn1 activates transcription and translation of mRNAs encoding membrane proteins
Source: Nat Commun. 2019 Mar 21;10:1298. doi: 10.1038/s41467-019-09199-6 (PMC6428865; doi:10.1038/s41467-019-09199-6)
Supplement: Supplementary file 1 — Supplementary Information [file 41467_2019_9199_MOESM1_ESM.pdf]

The exonuclease Xrn1 activates transcription and translation of mRNAs encoding membrane proteins

Blasco-Moreno et al.

SUPPLEMENTARY FIGURES

Supplementary Fig. 1

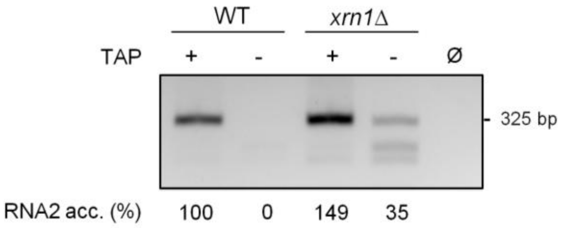

Supplementary Figure 1. A minor fraction (23%) of RNA2 molecules are uncapped in *xrn1*Δ cells. Agarose gel analysis of the PCR products obtained after RLM-RACE analysis of RNA prepared from both WT and *xrn1*Δ cells. Reactions were performed both with and without pre-treatment of the RNA with tobacco acid pyrophosphatase (+/−). Also shown is a negative control (Ø) in the absence of added template. RT-PCR was performed using a sense primer specific to the 5′ annealed RNA oligo and a gene-specific antisense primer. Source data are provided as a Source Data file.

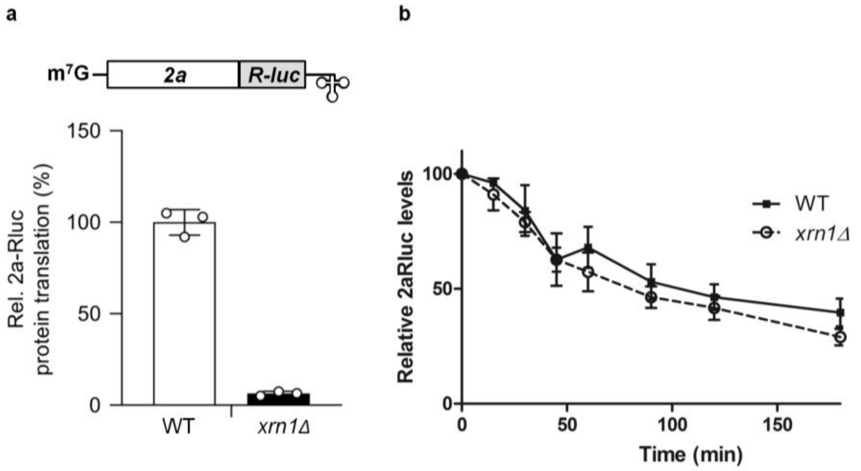

Supplementary Figure 2. The stability of BMV 2a protein is not affected in *xrn1*Δ yeast strain. a The RNA2-Rluc derivative depends on Xrn1 for translation. Top; schematic of the BMV RNA2-Rluc construct. It maintains the viral 5'UTR and 3'UTR and incorporates Renilla Luciferase (Rluc) in frame to the carboxy terminal of the 2a protein. Bottom; histogram showing the translation of 2a-Rluc (Light units/OD at 600 nm normalized by RNA2 accumulation measured by quantitative PCR) in wild-type (WT) and *xrn1*Δ strains. Open circles indicate the individual data points. **b** 2a-Rluc protein turnover is similar in WT and *xrn1*Δ strains. Cells were grown in log phase. The translation elongation inhibitor cycloheximide was added at a final concentration of 300 µg/ml. Samples were collected at different time points and measured for Rluc activity. The graph shows the normalized amount of 2a-Rluc (light units/OD at 600 nm) relative to the first time point. Results represent an average from n=3 biological replicates. Error bars depict SEM. Source data are provided as a Source Data file.

Supplementary Fig. 3

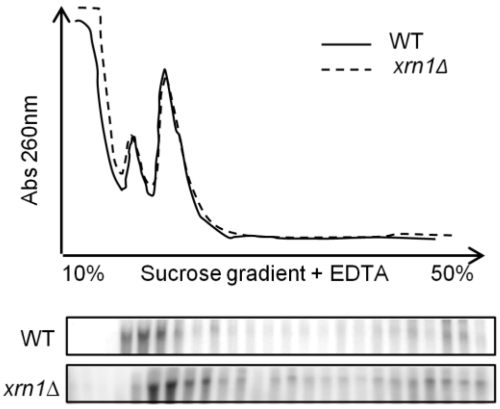

Supplementary Figure 3. EDTA treatment shifts RNA2 from heavy polysomes to lighter fractions. Top; UV absorbance at 260 nm corresponding to the polysome profile of WT and *xrn1*Δ strains after sedimentation on a 10 to 50% (wt/vol) sucrose gradient with 15 mM EDTA. Bottom; representative Northern blots corresponding to the BMV RNA2 present in each fraction. Source data are provided as a Source Data file.

Supplementary Fig. 4

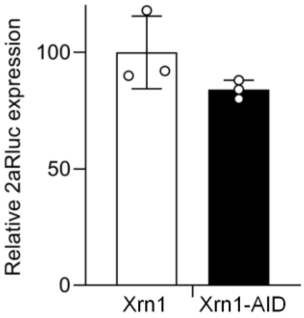

Supplementary Figure 4. Xrn1-AID fusion derivative promotes translation of BMV RNA2. Histogram depicts 2a-Rluc expression (light units/OD at 600 nm) in WT yeast cells and the isogenic strain with Xrn1 ORF fused to an auxin inducible degron (AID) in its C-terminus. Results represent an average from n=3 biological replicates. Error bars depict SEM. Open circles indicate the individual data points. Data source are provided as a Source Data file.

Supplementary Fig. 5

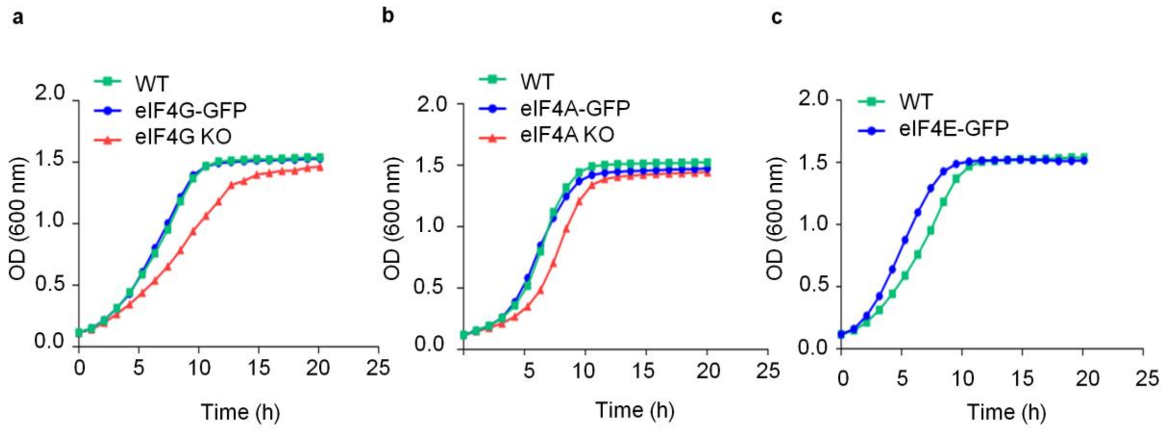

Supplementary Figure 5. GFP-fused eIF4G, -eIF4A and -eIF4E proteins are functional. Representative examples of growth curves at 30ºC for WT and (**a**) GFP-fused eIF4G strain and eIF4G knockout mutant (KO), (**b**) GFP-fused eIF4A strain and eIF4A KO mutant or (**c**) GFP-fused eIF4E strain. eIF4E KO mutant was not included because it is lethal. Source data are provided as a Source Data file.

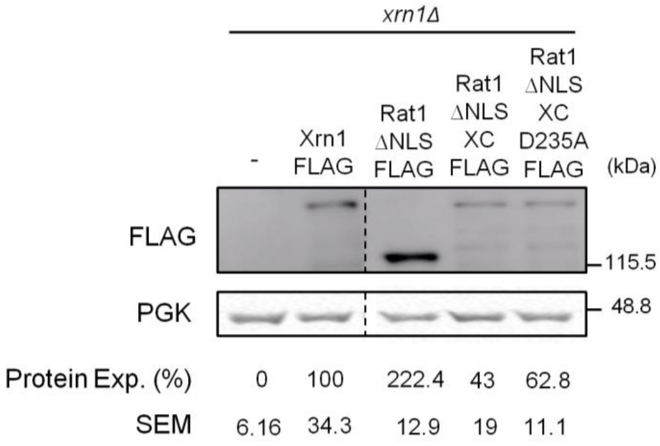

Supplementary Figure 6. Protein expression levels of FLAG-tagged Xrn1 and FLAG-tagged Rat1 derivatives analyzed by Western blot. Quantifications are relative to *xrn1*Δ transformed with WT Xrn1 plasmid. Results represent averages of n=3 biological replicates. Source data are provided as a Source data file.

Supplementary Fig. 7

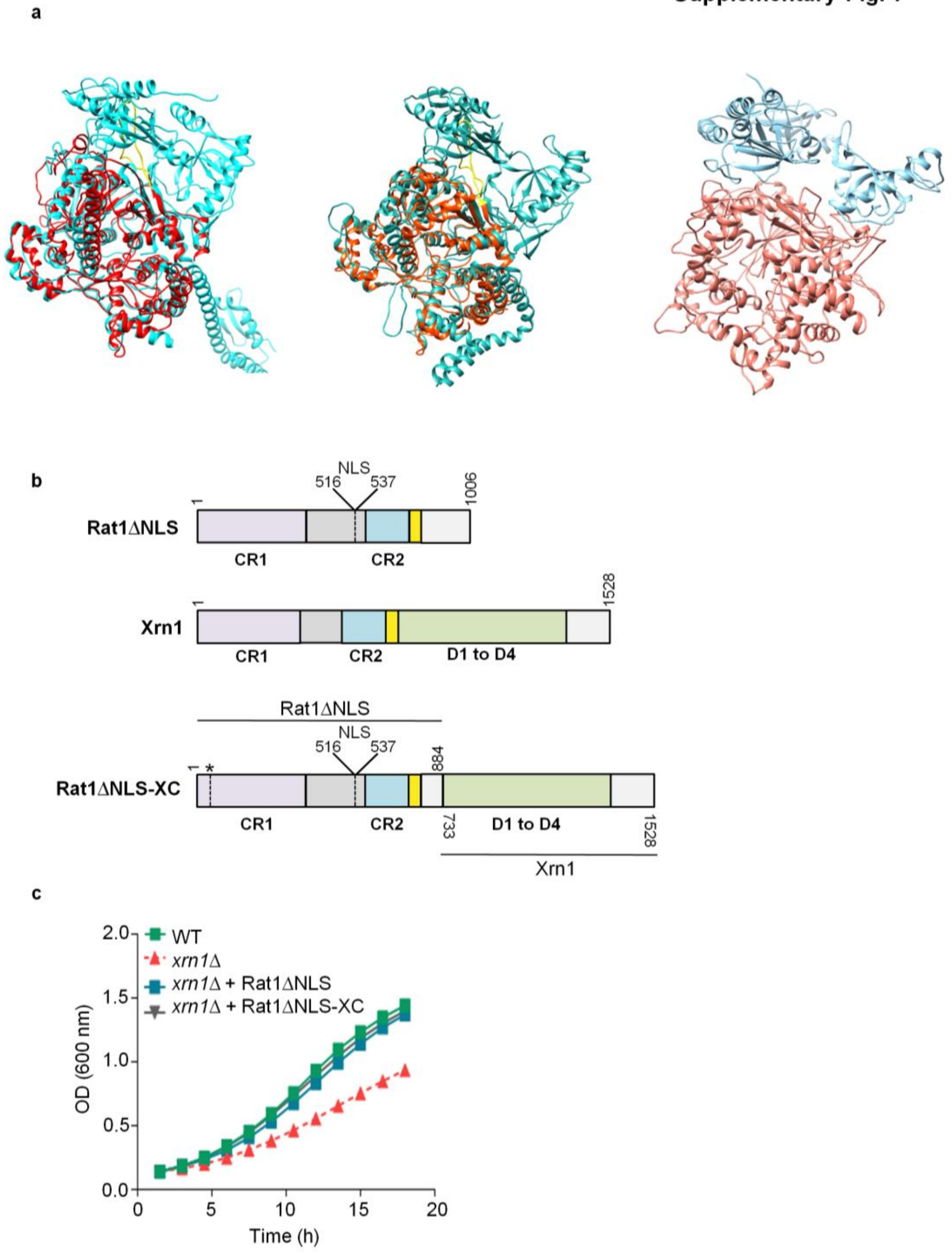

Supplementary Figure 7. Design of the Rat1-Xrn1 chimera (Rat1ΔNLS-XC). a Left: The superposition of PDB structures 3FQD (in red, for Rat1 in *S. pombe*) and 3PIF (in cyan, for Xrn1 in *K lactis*) show a colliding loop region (in yellow) hampering the interaction between the N-terminal and the C-terminal domains of a potential chimera.

Center: We built with MODELLER the structures of Rat1ΔNLS using 3FQD as template (in orange), and Xrn1 using 3PIF as template (in blue). After removing the loop regions without template, and superposing both structures, we corroborate that the sequence fragment LEEQPQIVDGVIL (in yellow) collides and hampers the interaction between the N-terminal and C-terminal domains of the potential chimera. Right: The model of the Rat1ΔNLS-XC chimera is shown in ribbon, with the C-terminal domain in cyan and the N-terminal domain in orange. **b** Schematic of Rat1ΔNLS, Xrn1 and Rat1ΔNLS-XC chimera showing the main protein domains. * represents the depletion of the colliding loop region (Δ24-42 amino acids). **c** Growth curves at 30ºC for *xrn1*Δ cells expressing the indicated proteins. Source data are provided as a Source Data file.

Supplementary Fig. 8

a

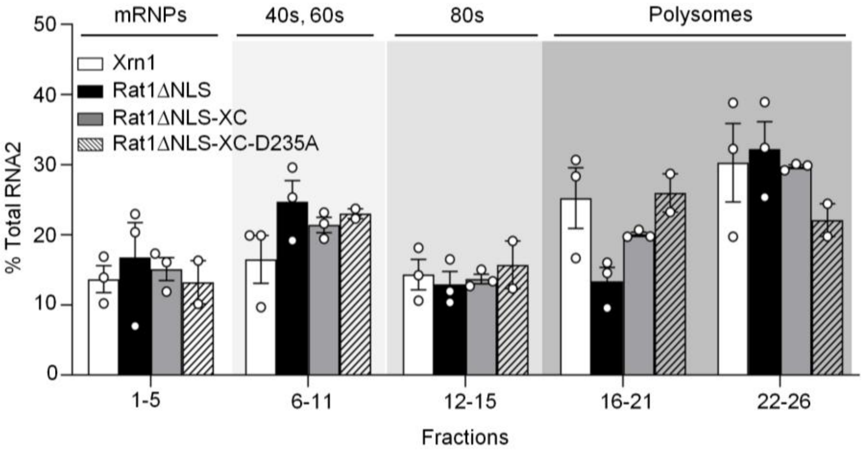

b

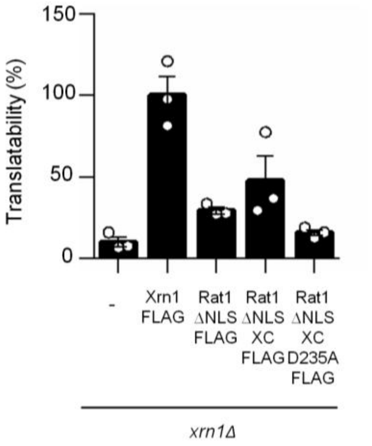

Supplementary Figure 8. Effect of Rat1 derivatives on translation of BMV RNA2. **a** Polysome profiling analyses of *xrn1*Δ cells expressing Xrn1, Rat1ΔNLS, Rat1ΔNLS-XC or Rat1ΔNLS-XC-D235A. Distribution of normalized BMV RNA2 accumulation across the profiles is shown. Fractions were grouped into free (1-5), single ribosome subunits (6-11), monosomes (12-15), light polysomes (16-21) and heavy polysomes (22-26). RNA was quantified by quantitative PCR. Results represent averages of n=3 biological replicates. Open circles indicate the individual data points. Error bars represent SEM. **b** Translatability values of RNA2 (change in 2a protein level divided by change in RNA2 level) in *xrn1*Δ cells expressing Xrn1, Rat1ΔNLS, Rat1ΔNLS-XC or

Rat1ΔNLS-XC-D235A. Quantifications are relative to *xrn1*Δ transformed with WT Xrn1 plasmid. Results represent averages of n=3 biological replicates. Open circles indicate the individual data points. Error bars represent SEM. Source data are provided as a Source Data file.

Supplementary Fig. 9

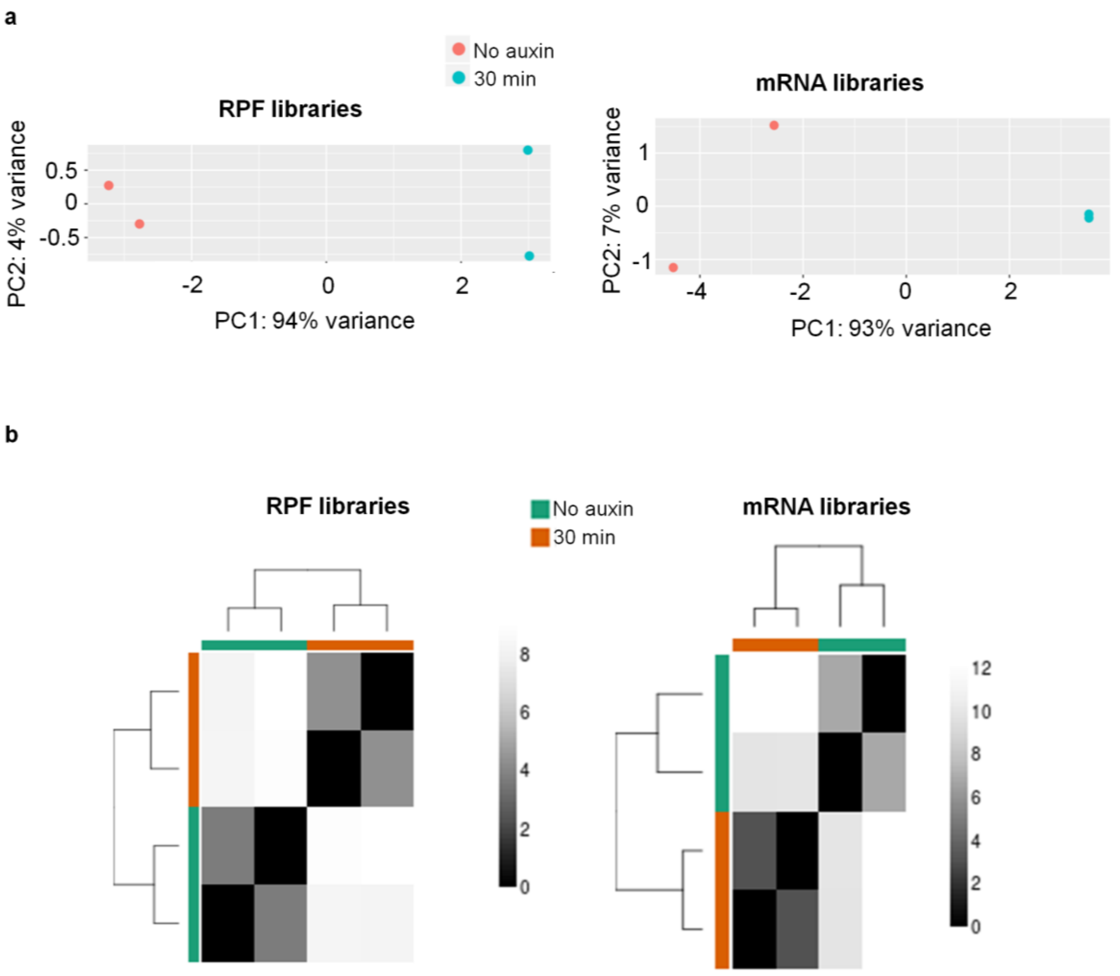

Supplementary Figure 9. Principal component analyses for RPF and RNAseq libraries showed clustering between replicates. a Principal Component (PC) analysis for RNAseq and RPF libraries for the two conditions: No auxin (red) and 30 minutes treatment (blue). **b** Similarity measurement between duplicates in RNAseq and RPF libraries.

Supplementary Fig. 10

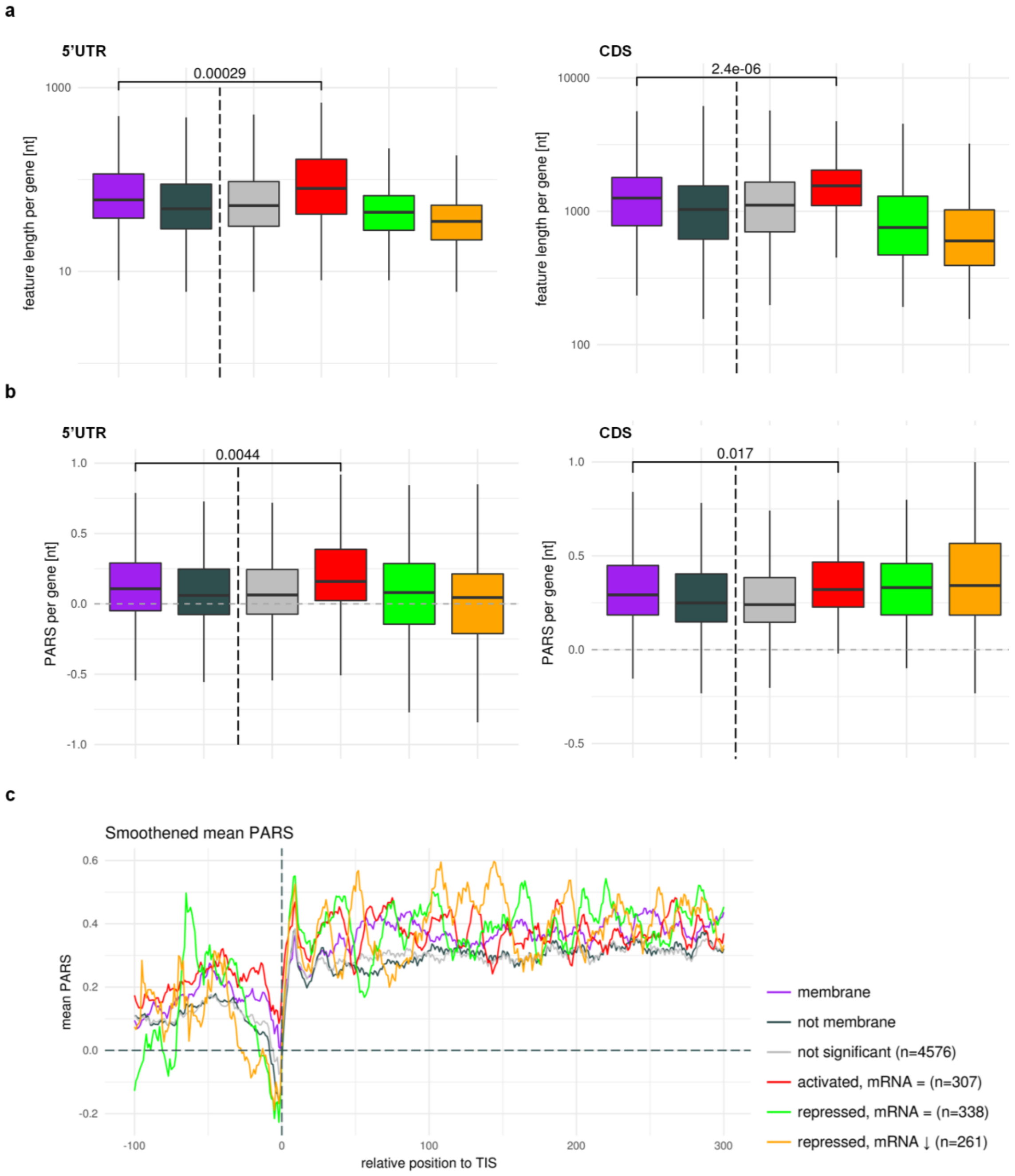

Supplementary Figure 10. Xrn1-activated mRNAs have longer and more structured 5'UTR and CDS in comparison to membrane mRNAs. a and **b** Box-plot depicting the mean length (**a**) and the mean PARS score (**b**) of the 5'UTRs and CDSs for the three groups studied, the control group and the mRNAs defined as "membrane"

and “not membrane”. For boxplots, box boundaries represent the 1st and 3rd quartile of the distribution, while the centre line represents the 2nd quartile (median). Whiskers indicate either the most extreme values or extend to 1.5 times the interquartile range starting from the respective box boundary.Statistical significance was calculated using a Wilcoxon-test. **c** Meta-gene analysis of the PARS scores. Vertical dashed line corresponds to the translation initiation site (TIS). The x-axis represents the nucleotide position relative to the TIS. The colour code detailed in **c** applies for all Supplementary Figure 10.

Supplementary Fig. 11

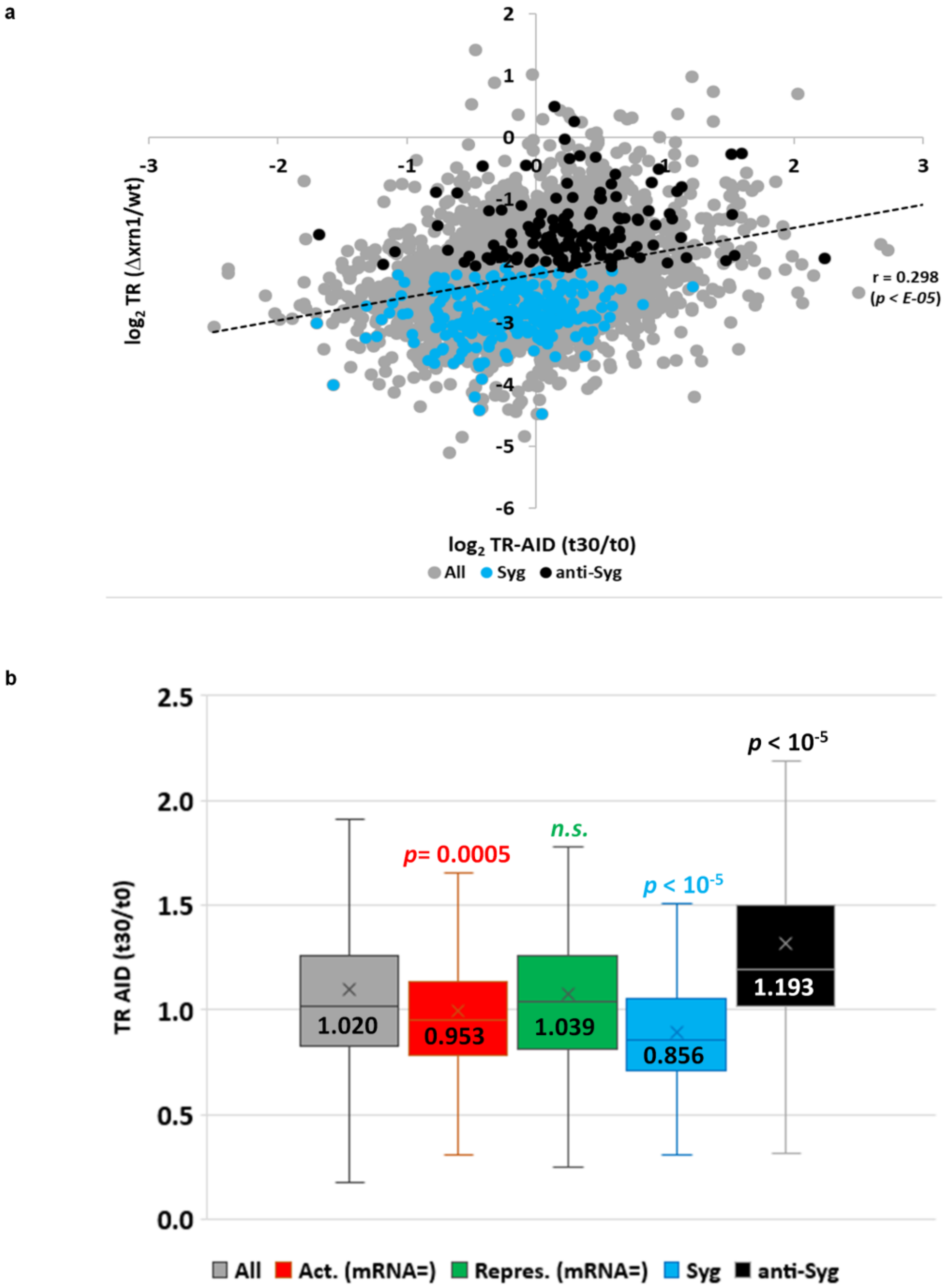

Supplementary Figure 11. Analysis of TRs in Xrn1-AID and *xrn1*Δ strains. a Double plot representing the change in TR upon depletion of Xrn1-AID (t30/t0) in the x-axis and the change in TR in xrn1Δ compared to WT (xrn1Δ/WT) in the y-axis. Genes

corresponding to “synthegradon” (Syg) and “antisynthegradon” (anti-Syg), as defined by[2], are represented. The effect in *xrn1*Δ is much stronger, but a shift in Syg and anti-Syg genes can be already observed in Xrn1-AID after 30 minutes of auxin treatment, anticipating the phenotype in *xrn1*Δ. **b** Change in TR upon Xrn1 depletion of our study groups (defined by Ribosome Profiling experiments) in comparison to Syg and anti-Syg. Statistical significance was calculated using a Wilcoxon-test. For boxplots, the bounds of the box represent the upper and lower quartiles (so the box spans the interquartile range). The centre line represents the median. Whiskers indicate the highest and lowest observations (values outside the whiskers are considered atypical values).

Supplementary Fig. 12

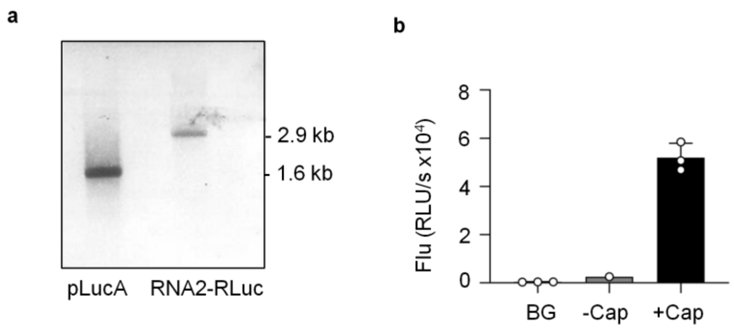

Supplementary Figure 12. Quality controls for electroporation experiment. a pLucA and RNA2-Rluc were *in vitro* transcribed and capped. Their relative size and integrity were analyzed with a denaturing formaldehyde gel. **b** As controls, yeast electroporation was performed without RNA (BG; background), with uncapped RNA (-Cap) and with capped RNA (+Cap). Results represent average of n=3 biological replicates. Error bars depict SEM. Open circles indicate the individual dot plots. Source data are provided as a Source Data file.
