# Supplementary material for: The exonuclease Xrn1 activates transcription and translation of mRNAs encoding membrane proteins
Source: Nat Commun. 2019 Mar 21;10:1298. doi: 10.1038/s41467-019-09199-6 (PMC6428865; doi:10.1038/s41467-019-09199-6)
Supplement: Supplementary file 4 — Description of Additional Supplementary Files [file 41467_2019_9199_MOESM4_ESM.docx]

**Description of Additional Supplementary Files**

File Name: Supplementary Data 1: Results of ribosome profiling analysis by Riborex and gene classification based on mRNA and RPF behaviour. Related to Fig. 5B.

Description: The columns represent: FDR – Benjamini-Hochberg-corrected p-value calculated by Riborex using both mRNA and RPF; relative_LFC – log_2_ fold change calculated by Riborex using both mRNA and RPF; LFC_mRNA_DESeq2 – DESeq2-derived log_2_ fold change for mRNA samples only; LFC_RPF_DESeq2 – DESeq2-derived log_2_ fold change for RPF samples only, class – gene classification based on mRNA and RPF data.

File Name: Supplementary Data 2: Enriched GO terms for selected gene classes. Related to Fig. 5C.

Description: The columns represent: domain – Gene ontology domain (BP – biological process, CC – cellular component, MF – molecular function), KEGG pathway (KEGG) or transcription factor (TF); p- value – Benjamini-Hochberg-corrected p-value calculated by gProfileR; term size – number of items related to a GO term; query size – number of valid genes used in database query; overlap size

- number of query genes belonging to GO term; term.id – GO term identifier; term.name – GO term description; REVIGO (Fig. 5C) – REVIGO-based classification of GO term redundancy, only “representative” terms were used for visualization in Fig. 5C.

File Name: Supplementary Data 3: Enrichment of membrane proteins among Xrn1-regulated transcripts. Related to Fig. 5D.

Description: The table contains results of Fisher's exact test comparing Xrn1- regulated transcripts against background (not significant) genes. Columns represent: gene class – group of genes tested for enrichment compared to background; membrane proteins – either all membrane genes or a specified sub-group defined by Ast et al. used for enrichment testing; p-value

- p-value assigned by fisher.test(); odds ratio – odds ratio obtained via fisher.test(), indicative of enrichment if >1 or depletion if <1. 95%; CI – 95% confidence interval for odds ratio.

File Name: Supplementary Data 4: Results of comparing structure and length of RNA features between different gene classes. Related to Fig. 6A.

Description: Sheet A (PARS) and B (featureLength): columns represent the name of the class used for comparison against genes that were “not significant”; rows contain corresponding log_2_ fold change (log_2_FC) and p-value (Wilcoxon-test), separate for individual RNA features (5' UTR, CDS, 3' UTR). Sheet C (mean PARS) and D (mean length): columns represent the name of the class, while rows contain mean or median length of individual RNA features for each class (5' UTR, CDS, 3' UTR).

File Name: Supplementary Data 5: Enriched GO terms using GSEA for the changes after Xrn1-KD inactivation. Related to Fig. 7A-B.

Description: Ratios t30/t0 in transcription rate, mRNA stability and mRNA level were analysed both for the highest (upper) and lowest (lower) ratios only in the biological process domain. Log of odds ratio (lor) and adjusted p-values given by the GSEA are shown.

File Name: Supplementary Data 6: Enriched GO terms and KEGG pathways for translationally activated genes (stable RNA levels) in Xrn1-D208A mutant. Related to Fig. 7C.

Description: Different gene ontology domains (BP – biological process, CC – cellular component, MF – molecular function) and KEGG pathways (KEGG) can be found in different sheets. Columns represent: p-value – Benjamini-Hochberg-corrected p-value calculated by gProfileR; term size – number of items related to a GO term; query size – number of valid genes used in database query; overlap size – number of query genes belonging to GO term; term.id – GO term identifier; term.name – GO term description.

File Name: Supplementary Data 7

Description: Yeast strains and plasmids used. Related to Methods.

File Name: Supplementary Data 8

Description: Sequences of primers used. Related to Fig. 5D and to Methods.

File Name: Supplementary Data 9

Description: List of antibodies used. Related to Methods.
