# Supplementary material for: The exonuclease Xrn1 activates transcription and translation of mRNAs encoding membrane proteins
Source: Nat Commun. 2019 Mar 21;10:1298. doi: 10.1038/s41467-019-09199-6 (PMC6428865; doi:10.1038/s41467-019-09199-6)
Supplement: Supplementary file 2 — Peer Review [file 41467_2019_9199_MOESM2_ESM.pdf]

Reviewers' comments:

Reviewer #1 (Remarks to the Author):

Review for the NCOMMS-18-22886 entitled "Xrn1 links 1 mRNA synthesis, translation and decay"

The highly conserved 5´-3´ exonuclease Xrn1 regulates gene expression in eukaryotes by coupling nuclear DNA transcription to cytosolic mRNA decay. In this study, the authors propose that Xrn1 acts as a translational modulator. Xrn1 promotes translation of a specific group of transcripts encoding membrane proteins that contain long and highly structured 5´UTRs. This regulation is mediated by a physical and functional interaction of Xrn1 with the translation initiation factor eIF4G and correlates with an Xrn1-dependence for mRNA localization at the endoplasmic reticulum. They also propose that for this group of mRNAs, Xrn1 stimulates transcription, mRNA translation, and decay. Finally, the authors suggest novel crosstalk between the three major stages of gene expression coordinated by Xrn1 to maintain appropriate levels of membrane proteins.
Their proposals are potentially interesting and relevant, however, the quality of results is not sufficient enough to support their proposal. The evidence to demonstrate the direct interaction between translation initiation factor eIF4G and Xrn1 and its functionally equivalent derivative is preliminary and lacks required controls. Overall, this study is potentially exciting but need more experimental evidence to be a candidate for the publication in Nature Communications. They give appropriate credits to previous work.

Comments
1. Evaluation of the role of Xrn1 in translation of Brome Mosaic virus RNA2 in yeast (Figure 2): Based on the distribution of RNA2 in fractions of sucrose-density gradients with wild-type and xrn1Δ mutant cell lysates, the authors propose that Xrn1-depletion shifts the RNA2 towards single ribosomal subunits fraction. The level of RNS2 was drastically increased in xrn1Δ mutant cells (4.6-fold, Figure 1a), and the decapped mRNAs are expected to be stabilized in the mutant cells to some extents but not translated. Therefore, it should be determined whether mRNAs in the mutant cells were capped or uncapped.

2. The interaction between Xrn1 and eIF4G and its functional relevance (Figure 4): Based on the results shown in Figure 4, the authors propose that the effect of Xrn1 on BMV RNA2 translation is specific and mediated by interactions with the translation machinery. However, the several essential controls were missing in the experiments. (i) The levels of immunoprecipitated GFP-fused eIF4G (Figure 4c and 4e), eIF4A (Figure 4c) or 4E (Figure 4c). (ii) The functional validation of GFP-fused eIF4G, eIF4A or 4E by the complementation of lethality of the mutant cells (Figure 4c).

3. C-terminal Xrn1 domain is crucial for translation activation of RNA2 (Figure 4d and e): The levels of RNA2 protein in xrn1Δ mutant cells were restored by Rat1ΔNLS-XC (Figure 4d), and Rat1ΔNLS-XC was co-immunoprecipitated with GFP-fused eIF4G. To evaluate the role of that C-terminal Xrn1 domain in translation activation of RNA2, it is needed to perform polysome analysis of RNA2 with lysates of xrn1Δ mutant cells expressing Xrn1, Rqt1ΔNLS, and Rat1ΔNLS-XC as shown in Figure 2. Besides, the direct interaction between eIF4G and C-terminal Xrn1 domain could be demonstrated with the binding assay with the recombinant proteins.

4. The linkage between mRNA transcription, decay, and translation in Xrn1 functions (Figure 7): Based on the results with Xrn1-D208A (Figure 7c), the authors propose that the functions of Xrn1 in transcription, translation, and decay are linked and require an active catalytic site. Since the levels of RNA2 protein in xrn1Δ mutant cells were restored by Rat1ΔNLS-XC (Figure 4d), the defects of the catalytically-inactive version of Rat1ΔNLS-XC in translation activation of RNA2 should be demonstrated by polysome analysis. The controls of the experiment shown in Figure 7d (electroporation in vitro transcribed RNA2-Rluc) were missing.

Reviewer #2 (Remarks to the Author):

Blasco-Moreno and colleagues here characterize a novel role of Xrn1 in translation control. They demonstrate Xrn1 activates translation of a BMV RNA2 reporter in a 5'UTR dependent manner. They show Xrn1 deletion results in a shift toward lighter polysome fractions and that Xrn1 associates with free 40S subunits. They then show that Xrn1 interacts with 4G, likely at the Xrn1 C-terminus, and that the Xrn1 C-terminus plays some role in translation activation. Blasco-Moreno et al. perform ribosome profiling and RNA-seq in Xrn1-depleted and WT cells to identify a subset of transcripts that are translationally activated and translationally repressed by Xrn1. Activated mRNAs in aggregate tend to have more structure in their 5' UTR. The manuscript argues that Xrn1 plays a specialized role in the translation and proper localization of transcripts encoding membrane proteins. They also characterize a catalytically-dead (D208A) Xrn1 and translation of transfected reporter mRNA to argue that Xrn1 links transcription, translation, and mRNA decay.

The manuscript provides a convincing argument for the novel role of Xrn1 in translation activation. Their work hints at a mechanism related to 5' UTR structure and interaction with eIF4G. I had a few major concerns related to the interpretation of the data and some of the conclusions.

1) The chosen title is not an accurate reflection of the provided data. Although the data detail the role of Xrn1 in translation, the proposed link between mRNA synthesis, translation, and decay requires further experimental support.

2) It is interpreted that Xrn1 plays a role in ER-localization of transcripts. Since ER-localized transcripts are dependent on translation for their ER-localization, an important alternative interpretation is that Xrn1 knock-down allows a pool of decapped and untranslated transcripts to persist in the cytoplasm.

I also had some minor concerns:

3) In figure 4, some Xrn1 C-terminal deletions and relevant point mutations would be more direct in characterizing the role of Xrn1 in translation activation. Some rescue is observed by Rat1ΔNLS (~3x in Fig 4d) and the further impact of Xrn1 C-terminus is limited (2x on top of Rat1ΔNLS alone).

4) A more-detailed methods write-up is missing for preparation and transfection of IVT RNA discussed in 7d. A supplementary figure to demonstrate IVT RNA is capped and is expected length can rule out possible technical artifacts.

5) Interpretation of 6d is as follows "…defects in elongation caused by ribosome stalling result in a peak in ribosome density. In contrast, differences in translation initiation result in a shift of ribosome occupancy along the entire CDS". Since the analysis is performed on a collection of transcripts that may have ribosome pausing/stalling in varying spots along the CDS, it does not necessarily follow that general reduction or increase along the CDS is a result of a translation initiation effect.

6) In supplementary figure 2, puromycin is more specific than EDTA to collapse polysomes.

7) In 3d, there is no distinguishable change in mRNA levels between + and - auxin, in contrast to what is reported in figure 1. Is this expected over the 50 min time course given that figure 4a suggests the RNA2 half-life is somewhere around 20 min?

Reviewer #3 (Remarks to the Author):

Evaluation of the manuscript "Xrn1 links mRNA synthesis, translation and decay" by Juana Diez and

co-workers.
In this manuscript, the authors describe a role of the cytoplasmic 5'-3' exonuclease Xrn1 in translation of mRNAs encoding membrane proteins in yeast. Xrn1 also enhances the localization of these mRNAs to the endoplasmic reticulum, where membrane proteins are translated. Furthermore, Xrn1 target mRNAs are characterized by highly structured 5' UTRs. Finally, the same transcripts that are translationally regulated by Xrn1 also depend on Xrn1 for transcription and decay, suggesting that the expression of membrane protein is regulated by Xrn1 at different steps of gene expression.
The manuscript describes an interesting phenomenon. However, some observations are difficult to explain, for example why Xrn1 has opposite effects on RNA2 mRNA translation depending on whether it is expressed from a plasmid or electroporated into the yeast cell (Fig. 1 vs. Fig. 7).
Additional comments:
1. My main concerns is the lack of a mechanism that could be responsible for the translation function of Xrn1. The weak interaction of Xrn1 with eIF4G is simply not sufficient to explain the strong effects.
2. Fig. 4c: A western blot for the GFP-tagged protein is missing and should be included.
3. Fig. 4b and d: what are the expression levels of Rat1 DelNLS and Rat1 DelNLS-XC compared to Xrn1?
4. Is it known if Rat1 DelNLS can rescue the transcription function of Xrn1? Since all the different functions of Xrn1 seem to be interconnected, it would be important to know that Rat1 DelNLS can rescue all functions but translation.
5. Fig. 5c: The p-value of the translationally repressed group with reduced mRNA (right panel) is the highest, while the other two groups (translationally activated and translationally repressed with =mRNA) are rather low. Is there any explanation for this observation? Wouldn't that mean that the translationally repressed group with reduced mRNA is the most significant?
6. Fig. 5d: the assay is not well described. Are the levels of the localized mRNAs normalized to the overall expression levels?

Response to comments by referee 1

1. Evaluation of the role of Xrn1 in translation of Brome Mosaic virus RNA2 in yeast (Fig. 2): Based on the distribution of RNA2 in fractions of sucrose-density gradients with wild-type and xrn1Δ mutant cell lysates, the authors propose that Xrn1-depletion shifts the RNA2 towards single ribosomal subunits fraction. The level of RNA2 was drastically increased in xrn1Δ mutant cells (4.6-fold, Fig. 1a), and the decapped mRNAs are expected to be stabilized in the mutant cells to some extents but not translated. Therefore, it should be determined whether mRNAs in the mutant cells were capped or uncapped.

We thank the reviewer for pointing out this issue. Previous results from the laboratory of Dr. Izaurralde demonstrated that efficient decapping requires the downstream 5´-3´exonuclease Xrn1 to associate with the decapping complex (Braun JE et al 2012; doi: 10.1038/nsmb.2413). This feedback mechanism assures that decapped mRNAs are immediately degraded. Consequently, in *xrn1Δ* mutant cells only a minor fraction of mRNAs are uncapped (Fig 1a; Hu W et al 2009; doi: 10.1038/nature08265). Indeed, we found similar results with RNA2. In the absence of Xrn1 only 23 % of RNA2 molecules are uncapped (Supplementary Fig. 1). We have now added this information in page 5.

2. The interaction between Xrn1 and eIF4G and its functional relevance (Fig. 4): Based on the results shown in Fig. 4, the authors propose that the effect of Xrn1 on BMV RNA2 translation is specific and mediated by interactions with the translation machinery. However, the several essential controls were missing in the experiments.
(i) The levels of immunoprecipitated GFP-fused eIF4G (Fig. 4c and 4e), eIF4A (Fig. 4c) or 4E (Fig. 4c).

Fig. 4c and 4e have been modified to include the requested controls.

(ii) The functional validation of GFP-fused eIF4G, eIF4A or 4E by the complementation of lethality of the mutant cells (Fig. 4c).

The functional validation of GFP-fused eIF4G, eIF4A and 4E are now shown in Supplementary Fig. 5.

3. C-terminal Xrn1 domain is crucial for translation activation of RNA2 (Fig. 4d and e): The levels of RNA2 protein in xrn1Δ mutant cells were restored by Rat1ΔNLS-XC (Fig. 4d), and Rat1ΔNLS-XC was co-immunoprecipitated with GFP-fused eIF4G. To evaluate the role of that C-terminal Xrn1 domain in translation activation of RNA2, it is needed to perform polysome analysis of RNA2 with lysates of xrn1Δ mutant cells expressing Xrn1, Rat1ΔNLS, and Rat1ΔNLS-XC as shown in Fig. 2.

This point is answered together with point 4 (see below).

4. The linkage between mRNA transcription, decay, and translation in Xrn1 functions (Fig. 7): Based on the results with Xrn1-D208A (Fig. 7c), the authors propose that the functions of Xrn1 in transcription, translation, and decay are linked and require an active catalytic site. Since the levels of RNA2 protein in

xrn1Δ mutant cells were restored by Rat1ΔNLS-XC (Fig. 4d), the defects of the catalytically-inactive version of Rat1ΔNLS-XC in translation activation of RNA2 should be demonstrated by polysome analysis.

We generated the catalytically-inactive version of Rat1ΔNLS-XC (Rat1ΔNLS-XC-D235A). As found for Xrn1, inactivation of the catalytic site within Rat1ΔNLS-XC inhibits RNA2 translation as shown by quantifying 2a protein and RNA2 levels (Supplementary Fig. 8b) and by polysome analyses. Polysome analysis of RNA2 included lysates of *xrn1Δ* mutant cells expressing Xrn1, Rat1ΔNLS, Rat1ΔNLS-XC and Rat1ΔNLS-XC-D235A (Supplementary Fig. 8a). The obtained results mirrored those obtained by quantifying 2a expression and RNA2 steady-state levels. When compared to expression of wild-type Xrn1, expression of Rat1ΔNLS shifts RNA2 from light polysome fractions to 40s and 60s fractions. In accordance with 2a translation experiments (Fig. 4d), this shift is indeed partially abrogated when Rat1ΔNLS-XC is expressed. In turn, expression of Rat1ΔNLS-XC-D235A induces a shift from heavy polysomes towards 40s and 60s fractions.

Besides, the direct interaction between eIF4G and C-terminal Xrn1 domain could be demonstrated with the binding assay with the recombinant proteins.

Xrn1 cannot be expressed in *E. coli*, which degrades it rapidly. Only truncated version of it could be detected (unpublished results). Our *in vivo* results in *S. cerevisiae* show that Xrn1 interacts with eIF4G in an RNase-independent manner, indicating that they interact directly or via other proteins. For clarity, we replaced the sentence in page 9 "…We conclude that Xrn1, but not Rat1ΔNLS **directly** interacts with eIF4G …" by "…We conclude that Xrn1, but not Rat1ΔNLS, interacts with eIF4G …"

The controls of the experiment shown in Fig. 7d (electroporation in vitro transcribed RNA2-Rluc) were missing.

We apologize for the omission. The corresponding controls showing the integrity and cap status of the RNAs (RNA2 and control pLucA) are now shown in Supplementary Fig. 12.

Response to comments by referee 2

1) The chosen title is not an accurate reflection of the provided data. Although the data detail the role of Xrn1 in translation, the proposed link between mRNA synthesis, translation, and decay requires further experimental support.

The title has been changed to "Xrn1 activates transcription, translation and decay of mRNAs encoding membrane proteins"

2**) It is interpreted that Xrn1 plays a role in ER-localization of transcripts. Since ER-localized transcripts are dependent on translation for their ER-localization, an important alternative interpretation is that Xrn1 knock-down allows a pool of decapped and untranslated transcripts to persist in the cytoplasm**

This is an interesting possibility. However, we consider it quite unlikely given that in the absence of Xrn1 only a minor fraction of mRNAs are decapped due to the requirement of Xrn1 for efficient decapping (Braun JE et al 2012; doi: 10.1038/nsmb.2413 and Fig 1a in Hu W et al 2009; doi: 10.1038/nature08265). Similarly, in *xrn1Δ* mutant cells only 23% of RNA2 molecules are uncapped (Supplementary Fig. 1).

Minor issues

In Fig. 4, some Xrn1 C-terminal deletions and relevant point mutations would be more direct in characterizing the role of Xrn1 in translation activation. Some rescue is observed by Rat1ΔNLS (~3x in Fig 4d) and the further impact of Xrn1 C-terminus is limited (2x on top of Rat1ΔNLS alone)

We agree with the referee that further analysis of the role of Xrn1 C-terminal region in translation will be of interest. However, we consider this beyond the scope of the current manuscript. Of note, previous studies have shown that deletions within the C-terminal residues result in a loss of the exonucleolytic activity (Page et al., 1998; doi.org/10.1093/nar/26.16.3707). Given that our results demonstrate that an active exonucleolytic site is important in Xrn1-dependent translational activation, it will be problematic and time consuming to uncouple effects on translation from those on exonucleolytic activity.

A more-detailed methods write-up is missing for preparation and transfection of IVT RNA discussed in 7d. A supplementary Fig. to demonstrate IVT RNA is capped and is expected length can rule out possible technical artifacts.

We apologize for the omission. The corresponding controls showing the integrity and cap status of the RNAs (RNA2 and control pLucA) are now shown in Supplementary Fig. 12 and a more-detailed method is now included in the methods section.

Interpretation of 6d is as follows "...defects in elongation caused by ribosome stalling result in a peak in ribosome density. In contrast, differences in translation initiation result in a shift of ribosome occupancy along the entire CDS". Since the analysis is performed on a collection of transcripts that may have ribosome pausing/stalling in varying spots along the CDS, it does not necessarily follow that

general reduction or increase along the CDS is a result of a translation initiation effect.

The reviewer raises a valid concern, however, even though a peak caused by ribosome stalling would not occur in the same relative position, the stalling would cause an accumulation of ribosomes upstream of the stalled ribosome, which would in turn lead to an increased ribosome footprint density upstream of the stalling site. Therefore, if a general elongation defect were to occur, one would expect to observe a change in the slope of the footprint density of the metagene, with the 5' end showing an increase and the 3' end showing a decrease in footprint density. Since this is not the case and we instead see virtually identical profiles that are shifted after Xrn1-KD, we conclude the defect has to occur during translation initiation.

To clarify this point, we rewrote the corresponding section to read: "...defects in elongation caused by ribosome stalling result in a peak in ribosome density **and an accumulation of ribosomes upstream of the stalling site. This would be visible as a change in slope in a metagene analysis, with the 5' end showing an increased footprint density and the 3' end showing a decrease.** In contrast, differences in translation initiation result in a shift of ribosome occupancy along the entire CDS"

6) In supplementary Fig. 2, puromycin is more specific than EDTA to collapse polysomes.

We thank the referee for the suggestion. Unfortunately, wild-type *S. cerevisiae* is insensitive to the effects of puromycin due to poor drug uptake. A mutant strain lacking Erg6, Pdr1, and Pdr3 is sensitive to puromycin , however it has delayed growth and is refractory to transformation by standard methods (Cary GA et al 2014, Yeast; doi:10.1002/yea.3007). Furthermore, we could not detect any release of elongating ribosomes from mRNAs even when this mutant strain was grown in the presence of puromycin for extended periods of time (D.D. Nedialkova and S.A. Leidel, unpublished observations). Consequently, to the best of our knowledge, there are no well-established protocols for performing drug-based ribosome release experiments in yeast.

7) In 3d, there is no distinguishable change in mRNA levels between + and - auxin, in contrast to what is reported in Fig. 1. Is this expected over the 50 min time course given that Fig. 4a suggests the RNA2 half-life is somewhere around 20 min?

We have modified Fig. 3d to include a later time point in which levels of RNA2 are increased in auxin-treated samples. Our interpretation is that at 50 minutes after auxin addition steady-state levels have not been reached yet and defects in RNA2 degradation are compensated by defects in RNA2 transcription (as described in Haimovich et al. 2013 Cell; doi:10.1016/j.cell.2013.05.012).

Response to comments by referee 3

... some observations are difficult to explain, for example why Xrn1 has opposite effects on RNA2 mRNA translation depending on whether it is expressed from a plasmid or electroporated into the yeast cell (Fig. 1 vs. Fig. 7).

These opposite effects suggest a linkage between the function of Xrn1 in transcription and translation. If Xrn1-dependent translation requires a previous nuclear function of Xrn1 in transcription, electroporating RNA2 directly into the cytosol would result in an Xrn1-independent translation of RNA2. In other words, electroporating naked RNA2 uncouples the cytoplasmic function of Xrn1 from its nuclear function.

Additional: comments:

1. My main concern is the lack of a mechanism that could be responsible for the translation function of Xrn1. The weak interaction of Xrn1 with eIF4G is simply not sufficient to explain the strong effects.

As indicated in the discussion, multiple observations are consistent with a role of Xrn1 in translation initiation. These include polysome profiling analyses (Fig. 2), ribosome profiling analyses (Fig. 6d), and the genetic (Sinturel F et al 2015; doi: 10.1016/j.celrep.2015.08.016), physical and functional interaction of Xrn1 with eIF4G (Fig. 4c-d). We agree with the reviewer that other factors are likely involved in the function of Xrn1 in translation and it is an interesting question to pursue in the future. However, we consider it out of the scope of the current manuscript.

2. Fig. 4c: A western blot for the GFP-tagged protein is missing and should be included.

Fig 4c has been modified to include the GFP-tagged proteins.

3. Fig. 4b and d: what are the expression levels of Rat1ΔNLS **and** Rat1ΔNLS-XC **compared to Xrn1?**

We thank the referee for pointing out this omission. Xrn1, Rat1ΔNLS and Rat1ΔNLS-XC proteins were tagged with an identical FLAG-tag. Expression levels of Xrn1, Rat1ΔNLS and Rat1ΔNLS-XC are similar, except for Rat1ΔNLS, whose expression level is even higher that the others (Supplementary Fig. 6). Therefore, the effects of Rat1ΔNLS and Rat1ΔNLS-XC on RNA2 translation cannot be explained by differential expression of Xrn1, Rat1ΔNLS or Rat1ΔNLS-XC.

4. Is it known if Rat1ΔNLS can rescue the transcription function of Xrn1? Since all the different functions of Xrn1 seem to be interconnected, it would be important to know that Rat1ΔNLS can rescue all functions but translation.

Rat1ΔNLS has the nuclear localization signal deleted and is localized in the cytoplasm. Therefore, unfortunately, we cannot measure its possible effects on transcription.

5. Fig. 5c: The p-value of the translationally repressed group with reduced mRNA (right panel) is the highest, while the other two groups (translationally activated and translationally repressed with =mRNA) are rather low. Is there any explanation for this observation? Wouldn't that mean that the translationally repressed group with reduced mRNA is the most significant?

The individual p-values of a GO term analysis are strongly dependent on the size and homogeneity of the group. The group of Ribosomal Proteins (and in lesser extent the groups Ribosome Biogenesis and Translation factors) are groups of some hundreds of genes that have a very homogeneous behavior in all GO analyses. Both features contribute significantly to produce extremely low p-values. Moreover, this analysis reflects the enrichment of genes associated to a particular term within the tested group compared to the rest of the genome and is not indicative of the importance of individual terms between different groups. For instance, it is possible to see enrichment of general / unspecific terms (such as "transcription, DNA-templated") with very low p-values ($p < 0.0000001$) in one group, while another group might be significantly enriched for genes associated to "mitochondrial ATP synthesis coupled electron transport" and "mitochondrial electron transport, cytochrome c to oxygen" with $p < 0.0001$, two terms that are highly specific and have fewer genes associated to them. Despite the lower p-value found for group 1, group 2 will be of higher interest as we can see association to a specific biological process that can be studied in further detail. Lastly, we would like to emphasize that Fig 5c is showing a collapsed version of Table **S2,** in which related terms have been grouped by REViGO to allow an easier visualization. For clarity, this is now stated in the corresponding methods section (page 47).

6. Fig. 5d: the assay is not well described. Are the levels of the localized mRNAs normalized to the overall expression levels?

The same number of WT and *xrn1Δ* cells was used for fractionation experiments. The ratio of specific mRNAs between supernatant and membrane was calculated in WT and *xrn1Δ* conditions. Xrn1-dependence for ER localization was calculated by dividing $\frac{\left(\frac{cc}{cm}\right)xrn1\Delta}{\left(\frac{cc}{cm}\right)WT}$ (cc = conc. in the cytosol fraction; cm =concentration in the membrane fraction). In Fig. 5d we represent for each mRNA these values relative to that of *TUB2,* which was set to 1. For clarity we specified this now in the corresponding figure legend. We would like to thank reviewer 3 for helping us to clarify this point.

REVIEWERS' COMMENTS:

Reviewer #1 (Remarks to the Author):

Review for the revised NCOMMS-18-22886A entitled “Xrn1 activates transcription, translation and decay of mRNAs encoding membrane proteins”

The authors have addressed most of my previous concerns. I support the publication of this revised MS in Nature Communications.

Reviewer #2 (Remarks to the Author):

The revised manuscript has addressed many concerns.

However, questions still remain about the role for uncapped mRNA accumulation and its apparent effect on translation.

Supplementary Figure 1 does seem to indicate the accumulation of uncapped RNA2, both full-length along with decay products (not clear whether these are included in the quantification). Thus, these results do point to a role for an accumulation of uncapped mRNAs and partial decay products — which are ascertained by mRNA sequencing but not translated — in changing measured translational efficiencies in xrn1Δ cells.

In considering the rebuttal, Hu & Coller 2009 Figure 1a show that xrn1Δ accumulates uncapped RNA, consistent with the effect that we would expect following the loss of the major 5’-to-3’ exonuclease. While this decapped mRNA can be associated with polyribosomes, the full-length capped mRNA is more strongly polyribosome associated. Hu and Coller broadly argue that decapping can occur while an RNA is being translated and propose that this decapping blocks further translation initiation. More recent work has demonstrated co-translational 5’-to-3’ decay on these ribosome-associated, decapped transcripts (Pelechano + Steinmetz, Cell 2015), which is presumably reduced in xrn1Δ.

Braun et al. study specifically GW182-induced decapping, which is not directly relevant to S cerevisiae, which lack a GW182 protein and indeed the entire RNA-induced silencing machinery.

It seems important to address the possibility of untranslatable mRNA/fragment accumulation for endogenous genes in Figure 5, particularly in light of the fact that structured 5’ UTRs are likely to impede decay of uncapped mRNAs.

Reviewer #3 (Remarks to the Author):

The authors addressed all my comments and improved the manuscript considerably. In my opinion it can now be published without further modifications.
